# The Brescia internationally validated European guidelines on minimally invasive liver surgery

**DOI:** 10.1093/bjs/znaf113

**Published:** 2025-06-26

**Authors:** Mohammad Abu Hilal, Tijs J Hoogteijling, Bjørn Edwin, Ibrahim Dagher, Mathieu D'Hondt, Hugo P Marques, Rutger-Jan Swijnenburg, Ugo Boggi, Iswanto Sucandy, Alessandro Ferrero, Hendrik Marsman, Roberto I Troisi, Andrew A Gumbs, Luca A Aldrighetti, Daniel Cherqui, Adnan Alseidi, Roland Croner, Fernando Rotellar, Olivier Scatton, Hani Al Saati, Giammauro Berardi, Umberto Cillo, Federica Cipriani, Ruben Ciria, Esteban Cugat, Fabrizio Di Benedetto, Rafael Diaz-Nieto, Trish Duncan, Mikhail Efanov, Giuseppe M Ettore, Constantino Fondevila, Asmund A Fretland, Felice Giuliante, Jeroen Hagendoorn, Ho-Seong Han, Goro Honda, Mickael Lesurtel, Peter Lodge, Santiago Lopez Ben, Riccardo Memeo, Krishna Menon, Niki Rashidian, Francesca Ratti, Ricardo Robles Campos, Nadia Russolillo, Andrea Ruzzenente, Julio Santoyo, Moritz Schmelzle, Olivia Sgarbura, Robert P Sutcliffe, Catherine S C Teh, Marco Vivarelli, Go Wakabayashi, Steven White, Neil Pearce, Roberta Angelico, Elisa Bannone, Andrea Benedetti Cacciaguerra, Emre Bozkurt, Maria Conticchio, Soufyan El Adel, Simone Famularo, Martina Guerra, Christoph Kuemmerli, Victor Lopez-Lopez, Fernando Pardo Aranda, Gabriela Pilz da Cunha, Florian Primavesi, Sara Saeidi, Andrea R G Sheel, Jasper P Sijberden, Francesco Sucameli, Francesco Taliente, Teodros Veronesi, Jawad Ahmad, Sergio Alfieri, Fabio Ausania, Gianluca Baiocchi, Stefano Berti, Marc G Besselink, Salam Daradkeh, Luciano De Carlis, Antonella Delvecchio, Christos Dervenis, Safi Dokmak, Clarissa Ferrari, Giuseppe Kito Fusai, David Geller, Brian K P Goh, Alfredo Guglielmi, David Iannitti, Derar Jaradat, Elio Jovine, Chen Kuo-Hsin, Rong Liu, Ravi Marudanayagam, Federico Mocchegiani, Fabrizio Panaro, Antonio Pinna, Nazario Portolani, Omar Saleh, Claudio Sallemi, Alejandro Serrablo, Sameer Smadi, Amal Suhool, Mark A Taylor, Thomas M van Gulik, Ajith K Siriwardena, Horacio J Asbun

**Affiliations:** Department of Surgery, The University of Jordan, School of Medicine, Amman, Jordan; Department of Surgery, Amsterdam UMC Location University of Amsterdam, Amsterdam, The Netherlands; Cancer Center Amsterdam, Amsterdam, The Netherlands; The Intervention Centre and Department of HPB Surgery, Oslo University Hospital and Institute of Medicine, University of Oslo, Oslo, Norway; Department of Digestive Minimally Invasive Surgery, Antoine Béclère Hospital, Paris, France; Department of Digestive and Hepatobiliary/Pancreatic Surgery, Groeninge Hospital, Kortrijk, Belgium; Department of General Surgery, Centro Hospitalar Unversitario de Lisboa Central, Lisbon, Portugal; Department of Surgery, Amsterdam UMC Location University of Amsterdam, Amsterdam, The Netherlands; Cancer Center Amsterdam, Amsterdam, The Netherlands; Division of General and Transplant Surgery, University of Pisa, Pisa, Italy; Digestive Health Institute, AdventHealth Tampa, Tampa, Florida, USA; Department of General and Oncological Surgery, Umberto I Mauriziano Hospital, Turin, Italy; Department of Surgery, Onze Lieve Vrouwe Gasthuis, Amsterdam, The Netherlands; Department of Clinical Medicine and Surgery, Federico II University Naples, Naples, Italy; Department of Digestive Minimally Invasive Surgery, Antoine Béclère Hospital, Paris, France; Department of General, Visceral, Vascular and Transplant Surgery, University Hospital Magdeburg, Magdeburg, Germany; Hepatobiliary Surgery Division, IRCCS San Raffaele Hospital, Milan, Italy; Faculty of Medicine, Vita-Salute San Raffaele University, Milan, Italy; Hepatobiliary Center, Paul Brousse Hospital—Paris Sud University, Villejuif, France; Department of Surgery, Virginia Mason Medical Center, Seattle, Washington, USA; Department of Surgery, University of California San Francisco, San Francisco, California, USA; Department of General, Visceral, Vascular and Transplant Surgery, University Hospital Magdeburg, Magdeburg, Germany; HPB and Liver Transplantation Unit, Department of Surgery, University Clinic, Universidad de Navarra, Pamplona, Navarra, Spain; Institute of Health Research of Navarra (IdisNA), Pamplona, Spain; Department of Digestive, HBP and Liver Transplantation Surgery, Pitié-Salpêtrière Hospital, AP-HP, Paris, France; Department of Surgery, Salmanyia Medical Complex, Manama, Bahrain; Division of General Surgery and Liver Transplantation, S. Camillo Forlanini Hospital, Rome, Italy; Department of Surgical, Oncological and Gastroenterological Sciences, General Surgery 2, Hepatopancreatobiliary Surgery and Liver Transplantation, Padua University Hospital, Padua, Italy; Hepatobiliary Surgery Division, IRCCS San Raffaele Hospital, Milan, Italy; Faculty of Medicine, Vita-Salute San Raffaele University, Milan, Italy; Unit of Hepatobiliary Surgery and Liver Transplantation, University Hospital Reina Sofia, Cordoba, Spain; Department of Surgery, Hospital Universitari Germans Trias I Pujol, Barcelona, Spain; Department of Surgery, Hospital Mutua de Terrassa, Barcelona, Spain; Hepato-Pancreato-Biliary Surgery and Liver Transplantation Unit, University of Modena and Reggio Emilia, Modena, Italy; Department of Surgery, Cambridge University Hospitals NHS Foundation Trust, Cambridge, UK; Liver Unit, Department of General Surgery, University Hospital of Wales, Cardiff, UK; Department of Hepato-Pancreato-Biliary Surgery, Moscow Clinical Scientific Center, Moscow, Russia; Division of General Surgery and Liver Transplantation, S. Camillo Forlanini Hospital, Rome, Italy; Department of General and Digestive Surgery, Hospital Clinic, Barcelona, Spain; The Intervention Centre and Department of HPB Surgery, Oslo University Hospital and Institute of Medicine, University of Oslo, Oslo, Norway; Department of Surgery, Fondazione Policlinico Universitario A. Gemelli IRCCS, Rome, Italy; Department of Surgery, Universitair Medisch Centrum Utrecht, Utrecht, The Netherlands; Department of Surgery, Seoul National University Bundang Hospital, Seoul National University College of Medicine, Seoul, South Korea; Department of Surgery, Institute of Gastroenterology, Tokyo Women's Medical University, Tokyo, Japan; Department of HPB Surgery and Liver Transplantation, AP-HP, Beaujon Hospital, DMU DIGEST, Clichy, France; Department of Surgery, Leeds Teaching Hospitals NHS Trust, Leeds, UK; Servei de Cirurgia General i Digestiva, Hospital Doctor Josep Trueta de Girona, Girona, Catalonia, Spain; Department of Hepato-Pancreatic-Biliary Surgery, ‘F. Miulli’ General Regional Hospital, Acquaviva delle Fonti, Bari, Italy; Department of Medicine and Surgery, LUM University, Casamassima, Bari, Italy; Department of Transplantation and Hepatopancreatobiliary Surgery, King's College Hospital NHS Foundation Trust, London, UK; Department of General and Hepatopancreatobiliary Surgery, Ghent University Hospital, Ghent, Belgium; Hepatobiliary Surgery Division, IRCCS San Raffaele Hospital, Milan, Italy; Faculty of Medicine, Vita-Salute San Raffaele University, Milan, Italy; Department of General, Visceral and Transplantation Surgery, Clinic and University Hospital Virgen de la Arrixaca, IMIB-ARRIXACA, El Palmar, Murcia, Spain; Department of General and Oncological Surgery, Umberto I Mauriziano Hospital, Turin, Italy; Department of Surgery, University of Verona, Verona, Italy; Department of General and Digestive Surgery, Vithas Hospital Malaga, Malaga, Spain; Department of General, Visceral and Transplant Surgery, Hannover Medical School, Hannover, Germany; Department of Oncologic Surgery, Regional Cancer Institute, Montpellier, France; Liver Unit, Queen Elizabeth Hospital, Birmingham, UK; Hepatobiliary Surgery Section, Makati Medical Center, Makati, The Philippines; HPB and Abdominal Transplantation Surgery, Department of Experimental and Clinical Medicine, University Hospital of Marche, Polytechnic University of Marche, Ancona, Italy; Department of Surgery, Ageo Central General Hospital, Ageo, Japan; Department of Surgery, Newcastle upon Tyne Hospitals NHS Foundation Trust, Newcastle upon Tyne, UK; PLANETS Cancer Charity, Emsworth, Hampshire, UK; Department of Surgical Science, HPB, and Transplant Unit, University of Rome Tor Vergata, Rome, Italy; Hepato Biliary Pancreatic (HPB) Surgery Unit, Pederzoli Hospital, Peschiera del Garda, Italy; HPB and Abdominal Transplantation Surgery, Department of Experimental and Clinical Medicine, University Hospital of Marche, Polytechnic University of Marche, Ancona, Italy; Department of General Surgery, Division of Hepatopancreatobiliary Surgery, Koc University Hospital, Istanbul, Turkey; Department of Hepato-Pancreatic-Biliary Surgery, ‘F. Miulli’ General Regional Hospital, Acquaviva delle Fonti, Bari, Italy; Department of Surgery, Amsterdam UMC Location University of Amsterdam, Amsterdam, The Netherlands; Cancer Center Amsterdam, Amsterdam, The Netherlands; Hepatobiliary Surgery Unit, Fondazione Policlinico Universitario A. Gemelli, IRCCS, Catholic University of the Sacred Heart, Rome, Italy; Department of Surgery, Poliambulanza Foundation Hospital, Brescia, Italy; Department of Visceral Surgery, University Hospital Basel, Basel, Switzerland; Department of General, Visceral and Transplantation Surgery, Clinic and University Hospital Virgen de la Arrixaca, IMIB-ARRIXACA, El Palmar, Murcia, Spain; Department of General and Digestive Surgery, Corachan Clinic, Barcelona, Spain; Department of Surgery, Amsterdam UMC Location University of Amsterdam, Amsterdam, The Netherlands; Cancer Center Amsterdam, Amsterdam, The Netherlands; Department of Visceral, Transplant and Thoracic Surgery, Medical University Innsbruck, Innsbruck, Austria; Department of Surgery, MASHHAD University of Medical Sciences, Mashhad, Iran; Department of Hepatobiliary and General Surgery, Royal Liverpool University Hospital, Liverpool, UK; Department of Surgery, Amsterdam UMC Location University of Amsterdam, Amsterdam, The Netherlands; Cancer Center Amsterdam, Amsterdam, The Netherlands; Department of Surgery, Poliambulanza Foundation Hospital, Brescia, Italy; Department of Surgery, Fondazione Policlinico Universitario A. Gemelli IRCCS, Rome, Italy; Universita degli Studi di Torino, Turin, Italy; Department of General Surgery, University Hospitals Coventry and Warwickshire, Coventry, UK; Department of Surgery, Fondazione Policlinico Universitario A. Gemelli IRCCS, Rome, Italy; Pancreato-Biliary and Robotic Surgery, HPB and Transplant Surgeon, Hospital Clinic, Barcelona, Spain; Department of Clinical and Experimental Sciences, University of Brescia, Brescia, Italy; Department of General and Oncological Surgery, Ferrero Hospital, Verduno, Italy; Department of Surgery, Amsterdam UMC Location University of Amsterdam, Amsterdam, The Netherlands; Cancer Center Amsterdam, Amsterdam, The Netherlands; Department of Surgery, The University of Jordan, School of Medicine, Amman, Jordan; Department of Surgery and Transplantation, Niguarda Hospital Milan & University of Milano-Bicocca School of Medicine and Surgery, Milan, Italy; Department of Hepato-Pancreatic-Biliary Surgery, ‘F. Miulli’ General Regional Hospital, Acquaviva delle Fonti, Bari, Italy; Department of Surgery, AGIA OLGA Hospital, Athens, Greece; Department of HPB Surgery and Liver Transplantation, AP-HP, Beaujon Hospital, DMU DIGEST, Clichy, France; Unit of Statistics, Istituto Ospedaliero Fondazione Poliambulanza, Brescia, Italy; HPB & Liver Transplant Unit, Royal Free London, London, UK; Department of Surgery, Division of Hepatobiliary and Pancreatic Surgery, University of Pittsburgh Medical Centre, Pittsburgh, Pennsylvania, USA; Department of Hepatopancreatobiliary and Transplant Surgery, Singapore General Hospital and National Cancer Centre Singapore, Singapore, Singapore; Surgery Academic Clinical Programme, Duke-National University of Singapore Medical School, Singapore, Singapore; Department of Surgery, University of Verona, Verona, Italy; HPB Surgery, Atrium Health, Charlotte, North Carolina, USA; Department of Surgery, Medical University of Graz, Graz, Austria; Department of Surgery, University Hospital Essen, Essen, Germany; Department of Surgery, IRCCS Azienda Ospedaliero-Universitaria di Bologna, Bologna, Italy; Department of Surgery, Far-Eastern Memorial Hospital, New Taipei City, Taiwan; Faculty of Hepatopancreatobiliary Surgery, The First Medical Center of Chinese People’s Liberation Army (PLA) General Hospital, Beijing, China; Liver Unit, Queen Elizabeth Hospital, Birmingham, UK; HPB and Abdominal Transplantation Surgery, Department of Experimental and Clinical Medicine, University Hospital of Marche, Polytechnic University of Marche, Ancona, Italy; Department of Surgery, Montpellier University Hospital, Montpellier, France; Department of Abdominal and Transplant Surgery, Cleveland Clinic Florida, Weston, Florida, USA; Department of Clinical and Experimental Sciences, Surgical Clinic, University of Brescia, Brescia, Italy; Independent Consultant and Practitioner Academic at LAP Research, Oxford, UK; Department of Intervention Radiology, Fondazione Poliambulanza Istituto Ospedaliero, Brescia, Italy; HPB Surgical Division, Miguel Servet University Hospital, Zaragoza, Spain; Department of Surgery, King Hussein Medical Center, Amman, Jordan; Department of Hepatopancreaticobiliary and Endocrine Surgery, Sheikh Shakhbout Medical City (SSMC), Abu Dhabi, United Arab Emirates; Department of Surgery, Mater Hospital, Belfast, Northern Ireland, UK; Department of Surgery, Amsterdam UMC Location University of Amsterdam, Amsterdam, The Netherlands; Cancer Center Amsterdam, Amsterdam, The Netherlands; Hepatobiliary and Pancreatic Surgery Unit, Manchester University NHS FT, Manchester, UK; Hepato-Biliary and Pancreas Surgery, Miami Cancer Institute, Miami, Florida, USA

## Introduction

The use of minimally invasive liver surgery (MILS) has increased in the last decades. Several studies have shown the benefits of laparoscopic liver resection (LLR) on short-term outcomes compared to conventional open surgery^1–3^ . In addition, since its introduction, robotic liver resection (RLR) is disseminating across specialized centres rapidly, producing excellent results and potentially promising further benefits in MILS^4^.

The introduction of MILS has been accompanied by several consensus-based meetings and evidence-based guidelines, to propose for careful implementation of MILS and ensuring patient safety. The first meetings were held in 2008 in Louisville and in 2015 in Morioka, followed by an increase in the application and the development of specialized equipment and techniques in MILS^5,6^. In 2017, the first European guidelines on LLR were developed in Southampton^7^. A total of 23 experts and seven researchers produced evidence-based guidelines on several domains related to LLR, adhering to the combination of several tried and tested methodologies of guideline formation. These guidelines set the standard for the safe development and dissemination of LLR, stressing the need for stepwise implementation.

Evolving experience and technical advancements such as RLR and artificial intelligence (AI) have caused a potential widening of the indications of MILS and a further increase in the application of minimally invasive techniques^8–12^. Since the Southampton guidelines, many studies have been published on MILS, including RCTs, large multicentre cohort studies, and meta-analyses. Furthermore, RLR is producing promising results, resulting in an unexpectedly rapid expansion. To ensure the safe further expansion of MILS, in which the improvement of outcomes and the minimization of risk are of the utmost importance, an update and reappraisal of the Southampton guidelines after more than seven years is necessary. Although LLR and RLR appear largely similar, important differences in instrumentation, surgical techniques, training programmes, and economic considerations require separate recommendations on either approach. Herein we present the results of the internationally validated European guidelines meeting on minimally invasive liver surgery.

## Methods

### Methodological framework

**Fig. 1:**
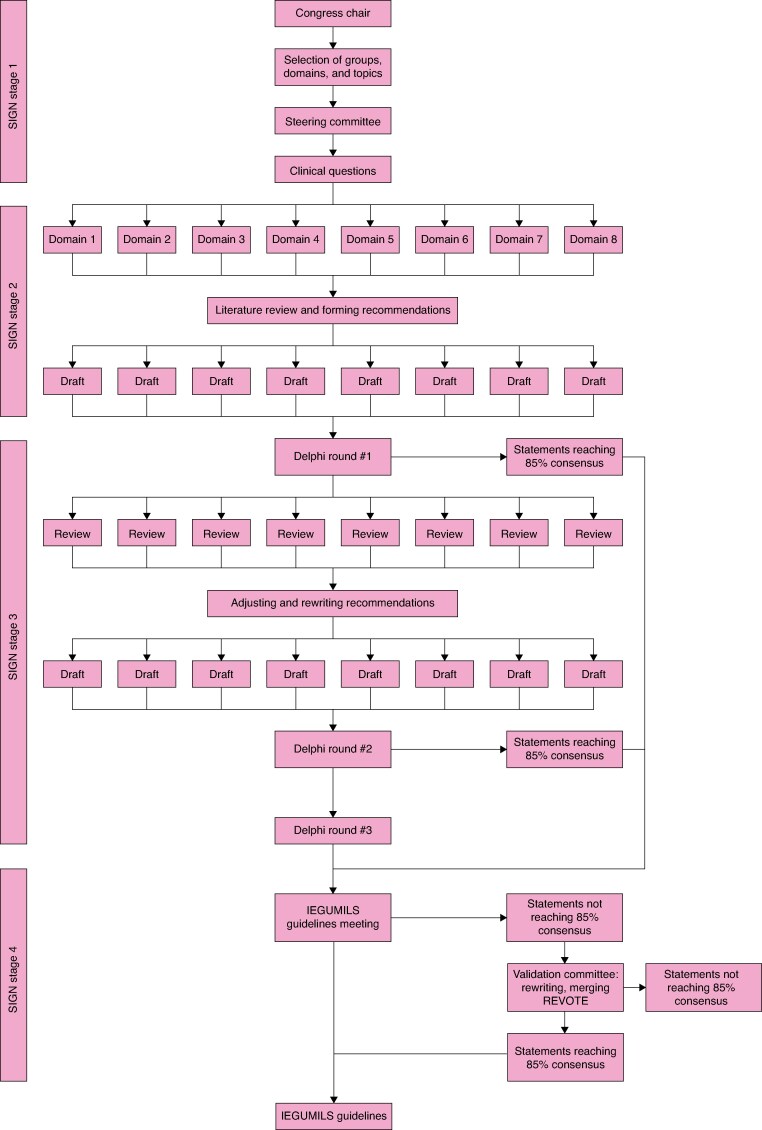
Guideline flowchart

To ensure evidence-based, transparent, and robust guideline development, the SIGN 50 (Scottish Intercollegiate Guidelines Network) was used as the methodological framework^13^. Following the SIGN 50, four stages were identified for the guidelines process: selection of groups, domains, and topics (stage 1); literature review and forming recommendations (stage 2); development of consensus (stage 3); consensus meeting and external review (stage 4). The guideline process is displayed in *Fig. 1*.

#### Stage 1: selection of committees

In the first stage, five different committees were established, and the congress chairman and steering committee selected members based on their experience in laparoscopic, robotic, and open liver surgery, as well as their scientific output. The steering committee consisted of eight members and the congress chair. This committee identified an expert committee of 37 European and eight international experts, a validation committee of 32 members including 30 liver surgeons, a methodologist and a patients’ representative, a jury committee of five members, and a research committee of 18 members dedicated to research in MILS (*Appendix S1*). The validation committee functioned as an independent quality assessment organ, as it did not participate in assessing the evidence and formulating recommendations. To ensure transparency in the preparatory work for the meeting, the chairman regularly updated the validation committee on the process.

After the group selection, eight key domains for guideline development were identified by the congress chairman and the steering committee, which included: terminology, indications, patient selection, procedures, surgical techniques and instrumentation, assessment tools, training and ICES (Implementation, Cost-Effectiveness, and Sustainability), and AI and innovative techniques. All domains were subsequently subdivided into 29 relevant topics, including clinical questions on laparoscopic, robotic, and general MILS, which were created and reviewed by the chairman and the steering committee.

#### Stage 2: working groups

During stage 2, the working groups of each domain conducted a systematic literature search of the available evidence on their topics, using online databases including PubMed, Embase, and Cochrane. A general search term for MILS in PubMed was created to standardize the literature search across all domains (*Appendix S2*). Queries for other terms and case-series and comparative studies including minimally invasive liver surgical procedures could be included. General exclusion criteria across all domains included not written in English, a sample size of fewer than 20 patients, and patient age under 18 years old (an exception was made for paediatric donor hepatectomies). All studies found eligible after screening were reviewed and summarized in summary of findings tables. Within the SIGN 50 framework, the GRADE (Grading of Recommendations, Assessment, Development and Evaluations) methodology was used to rate the quality of each study and assign a level of evidence (that is ‘high’, ‘moderate’, ‘low’, ‘very low’; *Appendix S3*)^14,15^. Based on the evidence and their quality, recommendations were formulated for each clinical question by the experts of the working group using the summary of findings tables and dedicated considered judgement forms (*Appendix S4*). In addition to the level of evidence, all recommendations were allotted a strength (that is ‘strong’ or ‘weak’; *Appendix S5*), following the GRADE methodology. Consequently, each recommendation was coded with a cipher and letter as follows: ‘1’ for strong recommendation, ‘2’ for weak recommendation, followed by ‘A’ for high level of evidence, ‘B’ for moderate level of evidence, ‘C’ for (very) low level of evidence. Each group delivered their final recommendations with a GRADE rating and the summary of findings tables to the congress chairman.

#### Stage 3: consensus development

In stage 3, consensus was created among the expert committee to ensure a broad base for the guidelines. In this phase the Delphi methodology was used to ensure anonymous, unbiased voting^16–18^. Experts could vote ‘agree’ or ‘disagree’ for each recommendation and could add a comment to each vote. Recommendations achieving an agreement rate of 85% or higher were approved. Those not achieving agreement were returned to their respective working groups for revision. After revision, the remaining recommendations were entered into a second Delphi voting round in which the voting process was repeated. Voting was done anonymously, and the comments on each question were only disclosed to their respective working groups. On 1 March 2024 and 3 April 2024, the first and second Delphi questionnaires were circulated among the experts. On 6 May 2024, prior to the official guideline meeting, a physical meeting with the chairman, steering, expert, and research committees was organized, in which a third and final Delphi round was held.

#### Stage 4: plenary in-person meeting

On 7–8 May 2024, as the fourth stage, the guidelines meeting took place in Brescia, Italy. At the start of the plenary meeting, professional oaths were sworn to the chairman by each committee leader, ensuring their commitment to an unbiased process (*Appendix S6*). During the two-day meeting, all evidence-based recommendations were presented by the domain working groups. The attending audience (consisting of residents, fellows, or surgeons who registered for the conference through the internationally validated European guidelines on minimally invasive liver surgery (IEGUMILS) website, as well as the expert and research committee members) voted ‘agree’ or ‘disagree’ using a digital voting device. The validation committee assessed the guideline process and quality for each topic according to the AGREE II-GRS instrument^19–23^ (*Appendix S7*). In private meetings after every two domain presentations, the validation committee further discussed the recommendations to propose alterations or mergers and subsequently assessed the English language quality. A separate jury committee consisted of validation committee members who were selected to validate and review the public voting process, evaluate and oversee the interaction between the expert and validation committee, and guarantee that the methodology was correctly followed. This jury completed a specifically designed form (*Appendix S8*) after each meeting day to assess quality aspects of the guidelines’ development process.

After the two-day meeting, the validation committee presented a report of their suggestions for adjustments or eliminations during a dedicated online meeting on 29 July 2024. All experts, researchers, and attendees of the IEGUMILS guidelines meeting were invited to attend and revote on the recommendations revised by the validation committee and those that initially did not achieve an audience agreement rate of 85%. All other adjustments and suggestions were reviewed and accepted by the chairman, steering committee, and expert committee.

## Results

**Fig. 2:**
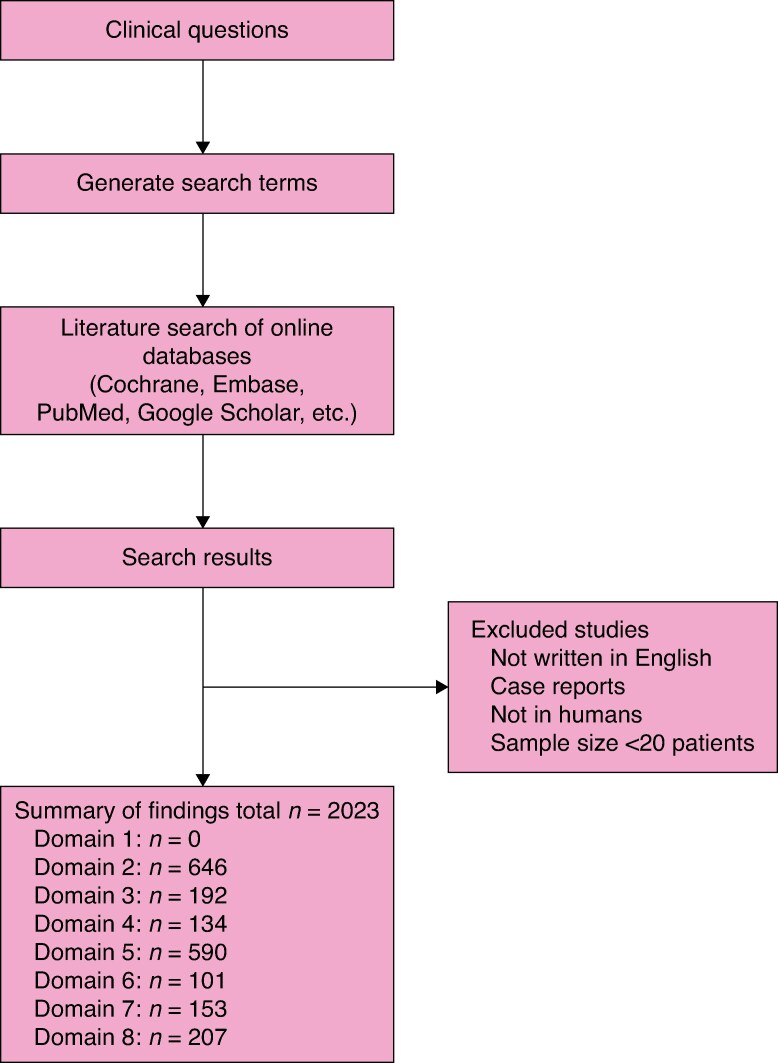
Literature search

The eight domains were subdivided into 29 topics. Each domain was assigned a working group consisting of between six and 12 experts, assisted by five or six researchers. The steering committee initially formulated 181 clinical questions: 75 on laparoscopic, 78 on robotic, and 28 on general MILS. In the guidelines process, 137 evidence-based recommendations were established: 44 for laparoscopic, 46 for robotic, 20 for combined laparoscopic and robotic, and 27 for general MILS. A flowchart of the literature search is shown in *Fig. 2*. The complete set of laparoscopic, robotic, and general clinical questions, recommendations and GRADE rating per domain and topic is provided in *Tables S1–8*. Expert and audience agreement rates, topic quality scores, comments, and literature are provided in *Table S9*.

### Domain 1: terminology

In domain 1, 11 definitions were established for the different types of minimally invasive surgical approaches, combined approaches, and conversions. The new set of agreed definitions of surgical approaches is shown in *Table 1*. Fully laparoscopic and robotic approaches are named ‘laparoscopic’ and ‘robotic’ respectively. The terminology for combined procedures contains both approaches, that is ‘combined robotic laparoscopic surgery’. The recommendations are shown in *Table S1*.

**Table 1:** Terminology in MILS

Terminology MILS	
There is no definition for ‘laparoscopic assisted’	
Laparoscopic surgery (LS)	The procedure is fully performed through laparoscopic ports.
Robotic surgery (RS)	The entire procedure is performed robotically. One or two assistant ports are permitted to assist the robotic procedure. The term is considered equivalent to robot-assisted surgery (RAS).
Combined robotic laparoscopic surgery (CRLS)	The procedure is performed through 4 robotic ports and 1 or more laparoscopic ports. The assistant is performing part of the surgery laparoscopically, beyond simply assisting the surgeon on the robotic console. Examples are when the bedside surgeon is using cavitronic ultrasonic surgical aspirator or energy devices.
Hand-assisted laparoscopy surgery (HALS)	The procedure is performed through laparoscopic ports and an auxiliary hand port. The procedure is performed laparoscopically, and one surgeon's hand is placed through the hand port and mostly used for retraction and palpation.
Hand-assisted robotic surgery (HARS)	The procedure is performed under robotic assistance and through robotic. An auxiliary hand port is also used. The procedure is performed robotically, and one surgeon's hand is placed through the hand port and mostly used for retraction and palpation.
Single-incision laparoscopic surgery (SILS)	The procedure is performed through a single incision using either standard or specific laparoscopic instruments. The use of an additional laparoscopic port (up to 5 mm) is permitted under the definition of single-incision laparoscopic liver surgery.
Single port robotic surgery (SPRS)	The procedure is performed through a single port using either standard or specific robotic instruments. The use of a laparoscopic port (up to 5 mm) is permitted under the definition of single-port robotic liver surgery.
Combined laparoscopic-open surgery (CLOS)*	A combined laparoscopic/open procedure, including a planned mini laparotomy that is performed for reasons different from specimen extraction.
Combined robotic-open surgery (CROS)*	A combined robotic/open procedure, including a planned mini laparotomy that is performed for reasons different from specimen extraction.
Transthoracic and/or transdiaphragmatic approach	A minimally invasive procedure performed through the chest. 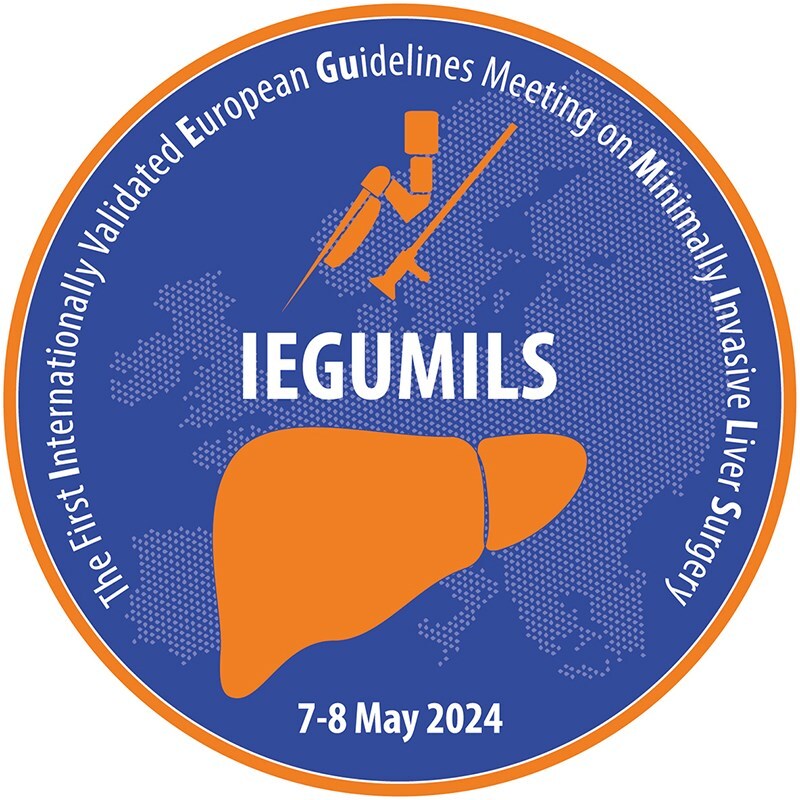

It is recommended that the new terminology should replace these terms. *Often these combined approaches were defined as ‘hybrid’ in prior publications.

### Domain 2: indications

In the domain ‘indications’, 18 recommendations were made on the various indications for MILS, including both malign and benign tumours, as well as living donor hepatectomies. Key recommendations for laparoscopic surgery are as follows: ‘LLR should be considered as a valid alternative approach to open surgery in the management of colorectal liver metastases (CRLM)’ (1A) and ‘When feasible, LLR should be preferred to open surgery in the management of hepatocellular carcinoma (HCC)’ (1B). Key recommendations for robotic surgery are as follows: ‘RLR offers similar short- and long-term results to LLR in the management of CRLM when performed in centres with adequate experience and expertise’ (2B) and ‘RLR for HCC is as safe and feasible as conventional open and laparoscopic approaches, offering comparable surgical and oncological outcomes in selected patients when performed in centres with adequate experience and expertise’ (2B). The full set of recommendations, including those on other indications for liver resection, is shown in *Table S2*.

### Domain 3: patient selection

In the domain ‘patient selection’, 21 recommendations were made on the safety and feasibility of MILS in different patient groups, including high-risk patients and patients undergoing technically complex resections. Patient age should not be considered a contraindication for both LLR (1B) and RLR (1C). Likewise, obesity should not be considered a contraindication for LLR (1B), but insufficient data were available regarding obese patients undergoing RLR. Key recommendations in this domain regard patients with cirrhosis: ‘LLR is indicated in patients with Child–Pugh A and is associated with some advantages compared to open resection, including less liver-specific complications. Pushing boundaries beyond Child–Pugh A still needs further evidence’ (1A) and ‘RLR is indicated in patients with Child–Pugh A and is associated with some advantages compared to open resection, including less liver-specific complications. Pushing boundaries beyond Child–Pugh A still needs further evidence’ (1C). The full set of recommendations is shown in *Table S3*.

### Domain 4: procedures

In the domain ‘procedures’, 9 recommendations were made on the application of MILS in minor, technically major, and major liver resections, as well as on anatomical and parenchyma-sparing liver resections. Key recommendations in this domain refer to laparoscopic minor and major liver resection: ‘LLR is safe for minor liver resections and a minimally invasive approach should be the preferred approach in the anterolateral segments’ (1B) and ‘LLR for right or left hemihepatectomy is technically feasible and safe in terms of morbidity and oncological outcomes’ (1B). The full set of recommendations is shown in *Table S4*.

### Domain 5: surgical techniques and instrumentation

In the domain ‘surgical techniques and instrumentation’, 20 recommendations were produced on techniques, tips and tricks, and instruments used specifically in MILS. The strongest recommendations in this domain regard the use of intraoperative ultrasound during liver resection: ‘Laparoscopic ultrasound should be available and considered in every case of LLR and RLR. Surgeons should be experienced in knowing the indications, performing and interpreting intra-operative ultrasound’ (1A and 1C for LLR and RLR respectively). The recommendations are shown in *Table S5*.

### Domain 6: assessment tools

In the domain ‘assessment tools’, four recommendations were produced on suitable outcome measurements of MILS, including clinical outcomes, composite outcomes, and patient reported outcomes (PROMs). The following recommendation was made for both LLR and RLR: ‘Intraoperative blood loss, operative time and hospital stay (days), conversion rate, R0/R1 resection rates and short-term clinical outcomes (mortality, overall complication rate, major complication rate (>grade 2 according to the Clavien–Dindo Classification), the Comprehensive Complication Index (CCI), failure to rescue rate each for 30 and 90 days after surgery) should be the core parameters in the assessment of LLR and RLR’ (1B). Additionally, the validation committee added a second statement regarding RLR: ‘Total costs should be defined as core in the assessment of RLR’ (1B), in recognition of both the direct and indirect costs involved with RLR. The full set of recommendations is shown in *Table S6*.

### Domain 7: training and implementation, cost-effectiveness, and sustainability

In the domain ‘training and ICES’, 19 recommendations were produced on the requirements of training programmes for surgeons performing MILS, and of the surgical centres in which MILS is being performed. It is recommended that ‘implementing structured training programmes with a step-up mastery progression is crucial in LLR and RLR, where advancement to the next phase is contingent upon mastery’ (1C), and that ‘surgeons intending to start an LLR or RLR practice should first pursue training through fellowships, courses, and a proctoring programme’ (1C). Two additional RLR recommendations were added: ‘Undertaking major robotic hepatectomy without sufficient RLR experience is strongly discouraged. The learning curve typically involves a minimum of 25 robotic minor hepatectomies’ (1C) and ‘LLR experience is not essential prior to starting RLR; however, it may reduce the learning curve’ (1C). A strong recommendation was made that ‘LLR should be adopted in all liver surgery centres and offered to patients with the appropriate indications according to the local level of proficiency’ (1A). In addition, recommendations on cost-effectiveness and sustainability were produced here. In summary, ‘the higher operative costs in LLR are offset by lower hospitalization costs (due to shorter hospital stay and less overall complications) compared to open liver resection, leading to no statistically significant difference in total costs in an RCT and a systematic review with economic analysis’ (1A). Given the limited data regarding the overall costs of RLR in comparison to the open approach, no definitive recommendations on the cost-effectiveness of RLR can be made. However, ‘Extended life programmes for robotic instruments and increased competition in the market should be encouraged to decrease costs for RLR’ (2C). The full set of recommendations is shown in *Table S7*.

### Domain 8: artificial intelligence and innovative techniques

In the domain ‘artificial intelligence and innovative techniques’, no validated recommendations were produced, due to very limited evidence and strong disagreement in the audience. Statements regarding the state of the art of AI and innovative techniques, as well as future requirements in research, are listed in *Table S8*.

## Discussion

The development of the IEGUMILS evidence-based guidelines followed stringent methodology and was overseen by established European and international experts and researchers in the field of minimally invasive and open liver surgery. These combined efforts have culminated in the production of 137 recommendations: 44 on laparoscopic, 46 on robotic, 20 on combined laparoscopic and robotic, and 27 on general MILS.

In the IEGUMILS guidelines, both LLR and RLR are considered indicated for the treatment of selected patients with (N)CLRM, HCC, intrahepatic cholangiocarcinoma, and benign liver tumours, and liver resections in the setting of living donor hepatectomy. This is remarkable because no previous guideline has clearly established the position of both LLR and RLR in the surgical treatment of all these indications simultaneously. The learning curve in minimally invasive surgery is recognized by the general addition to the recommendations that surgeons with adequate experience and expertise should perform MILS. Furthermore, surgeons who have undergone the correct training and have passed the learning curve are encouraged to perform major MILS, as well as complex resections in the posterosuperior segments or in complex groups of elderly and obese patients. These guidelines underline the trend towards a minimally invasive approach in liver surgery, striving for the best perioperative outcomes for patients, without sacrificing technical efficiency.

An important nuance regarding the widening of indications of MILS is that patient selection is crucial in MILS. Although these guidelines consider MILS indicated in almost all settings of liver disease, they do not render the open approach obsolete. An important concept in the interpretation and application of these guidelines is that the choice of surgical approach should depend on the skill and experience of the surgeon, not what is considered technically feasible in the literature. Weak recommendations based on low level of evidence serve more as an encouragement for those who have had proper training and are adequately experienced to push forward the boundaries of MILS. A controlled and safe setting is essential for this purpose. The recommendations by the IEGUMILS faculty do not authorize the careless application of minimally invasive techniques for the sake of experimentation but do in fact condemn it. The phrase ‘adequate experience and expert’ added to many statements might seem abstract at first but implies a responsible attitude towards applying MILS and recognizes the importance of proper training and practice. Hence, patient selection remains a vital aspect in MILS. The choice of surgical approach must consider the patient’s condition, the tumour’s characteristics, the extent of the resection, and the surgeon’s experience.

Compared to the Southampton guidelines, three new domains were added: terminology, outcome measurement and assessment tools, and artificial intelligence and innovative techniques. These added domains reflect the requirements of the MILS community, as well as the topics that recent research in MILS has covered. First, the use of uniform, internationally accepted terminology is essential in both clinical care and scientific research. The Brisbane 2000 terminology of liver resection determined that terminology should be: (linguistically) correct, consistent, self-explanatory, translatable, and precise^24^. Despite this universally accepted terminology on types of liver resection, there is a large variety on terminology of the minimally invasive approach. This variety is seen mostly in RLR and might be partly because surgeons use the robot in different ways during MILS—some performing the entire procedure with the robot, whereas others use the robot only for specific phases, or have the assistant perform certain manoeuvres with laparoscopic instruments. Consequently, several terms are used interchangeably in current literature, including ‘robotic’, ‘robot-assisted’, ‘robolap’, and ‘hybrid’. The IEGUMILS guidelines meeting has now produced a comprehensive set of definitions on laparoscopic, hand-assisted, robotic, and thoracoscopic approaches, which have been debated over and agreed on by the faculty experts and the public. Notably, the term ‘robotic’ should be used to indicate fully robotic procedures, in which one or two laparoscopic ports are allowed to assist the surgeon. When a robotic procedure is partly performed by laparoscopy, the term ‘combined robotic laparoscopic surgery (CRLS)’ should be used. This new terminology attempts to standardize reporting and provide a uniform system that can be used to better classify resections and compare outcomes.

Core outcome parameters were determined and are recommended to be used in reporting and registration of MILS. They include conventional perioperative and long-term survival outcomes, PROMs, and cost-effectiveness outcomes, which are recommended to better analyse the performance of minimally invasive approaches. Especially the measurement and reporting of total cost in RLR was considered an important parameter to be added to research of cost-effectiveness of MILS. In addition, composite outcome measures, including textbook outcome are recommended in outcome measurement of MILS, as well as benchmarking. This standardized outcome measurement is crucial to facilitate scientific research and facilitate the safe and efficient onward expansion of MILS.

The many recent technological innovations and the adoption of AI in liver surgery was covered in the last domain of IEGUMILS. Some of these techniques (that is indocyanine green) have established a place in routine practice, whereas others (including 3D reconstruction, automatic segmentation, and autonomous actions) are still considered experimental. Most of these innovative techniques are currently in their infancy, and their routine use cannot yet be recommended. Although promising results are being published on the use of AI, the level of evidence is generally very low. This final domain resulted in strong discussions among the faculty and public, and the general consensus was that it is too early to formulate evidence-based guidelines on this topic. Therefore, domain 8 was not validated by the validation committee. In recognition of the future prospects of AI and innovative techniques, the results of these discussions and general statements were included in the guidelines. The faculty of the IEGUMILS strongly feels that the future development of MILS will be parallelled and supported by these technical and digital innovations.

A more general but important finding of the IEGUMILS guidelines is that despite the scientific research on MILS, the level of evidence of many recommendations is relatively low. Moreover, in several domains, the evidence is limited to small case series or no evidence (classified as ‘very low’). These recommendations should be interpreted carefully, because clinical decision-making should primarily be evidence-based. However, despite the low level of evidence, a recommendation can nonetheless be strong. A good example is that even though no studies can be performed on haemodynamically unstable patients due to massive intra-abdominal haemorrhage, every surgeon would recommend performing an emergency conversion. In this case, the experts felt that although good-quality evidence was lacking, a strong recommendation could nevertheless be made. The objective here was to produce a practical guideline for the entire MILS community, not just a summary of all available literature. Guidelines are vital to the safe development and innovation of liver surgery^25^. In addition, IEGUMILS has identified important research gaps for future studies to focus on. These include the application of LLR and RLR in more extreme settings (that is second stage hepatectomy, or biliary/vascular reconstruction), cost-effectiveness of RLR, sustainability initiatives, and the application of artificial intelligence in liver surgery. In addition, future research should focus on laparoscopic *versus* robotic liver resection, not necessarily to investigate superiority, but rather to identify patients more suited to either laparoscopic or robotic resection. We are convinced that the laparoscopic and robotic approach should complement each other in contemporary and future MILS to achieve the best outcomes for patients. Selecting the right approach for the right patient should be a major theme in future research.

## Conclusion

The 2024 IEGUMILS meeting in Brescia resulted in 137 evidence-based recommendations on laparoscopic and robotic liver surgery, established by a group of recognized international and European experts in the minimally invasive and open liver surgery field. The Brescia guidelines provide the most up-to-date evidence and can provide evidence-based guidance to liver surgeons, policy makers, and patients.

## Supplementary Material

znaf113_Supplementary_Data

## Data Availability

This guideline is informed by a synthesis of existing evidence and expert consensus. No new data were generated or analysed in the development of this work.
