# Supplementary material for: The Brescia internationally validated European guidelines on minimally invasive liver surgery
Source: Br J Surg. 2025 Jun 26;112(6):znaf113. doi: 10.1093/bjs/znaf113 (PMC12199258; doi:10.1093/bjs/znaf113)
Supplement: znaf113_Supplementary_Data [file znaf113_supplementary_data.docx]

Supplementary Material

**The Brescia internationally validated European guidelines on minimally invasive liver surgery**

Mohammad Abu Hilal^1^*, Tijs J. Hoogteijling^2,3^*, Bjørn Edwin^4^, Ibrahim Dagher^5^, Mathieu D'Hondt^6^, Hugo P. Marques^7^, Rutger-Jan Swijnenburg^2,3^, Ugo Boggi^8^, Iswanto Sucandy^9^, Alessandro Ferrero^10^, Hendrik Marsman^11^, Roberto I. Troisi^12^, Andrew A. Gumbs^5,13^, Luca A. Aldrighetti^14,15^, Daniel Cherqui^16^, Adnan Alseidi^17,18^, Roland Croner^13^, Fernando Rotellar^19,20^, Olivier Scatton^21^, Hani Al Saati^22^, Giammauro Berardi^23^, Umberto Cillo^24^, Federica Cipriani^14,15^, Ruben Ciria^25^, Esteban Cugat^26,27^, Fabrizio Di Benedetto^28^, Rafael Diaz-Nieto^29^, Trish Duncan^30^, Mikhail Efanov^31^, Giuseppe M. Ettore^23^, Constantino Fondevila^32^, Asmund A. Fretland^4^, Felice Giuliante^33^, Jeroen Hagendoorn^34^, Ho-Seong Han^35^, Goro Honda^36^, Mickael Lesurtel^37^, Peter Lodge^38^, Santiago Lopez Ben^39^, Riccardo Memeo^40,41^, Krishna Menon^42^, Niki Rashidian^43^, Francesca Ratti^14,15^, Ricardo Robles Campos^44^, Nadia Russolillo^10^, Andrea Ruzzenente^45^, Julio Santoyo^46^, Moritz Schmelzle^47^, Olivia Sgarbura^48^, Robert P. Sutcliffe^49^, Catherine S.C. Teh^50^, Marco Vivarelli^51^, Go Wakabayashi^52^, Steven White^53^, Neil Pearce^54^, Roberta Angelico^55^, Elisa Bannone^56^, Andrea Benedetti Cacciaguerra^51^, Emre Bozkurt^57^, Maria Conticchio^40^, Soufyan El Adel^2,3^, Simone Famularo^58^, Martina Guerra^59^, Christoph Kuemmerli^60^, Victor Lopez-Lopez^44^, Fernando Pardo Aranda^61^, Gabriela Pilz da Cunha^2,3^, Florian Primavesi^62^, Sara Saeidi^63^, Andrea R.G. Sheel^64^, Jasper P. Sijberden^2,3^, Francesco Sucameli^59^, Francesco Taliente^33^, Teodros Veronesi^65^, Jawad Ahmad^66^, Sergio Alfieri^33^, Fabio Ausania^67^, Gianluca Baiocchi^68^, Stefano Berti^69^, Marc G. Besselink^2,3^, Salam Daradkeh^1^, Luciano De Carlis^70^, Antonella Delvecchio^40^, Christos Dervenis^71^, Safi Dokmak^37^, Clarissa Ferrari^72^, Giuseppe Kito Fusai^73^, David Geller^74^, Brian K.P. Goh^75,76^, Alfredo Guglielmi^45^, David Iannitti^77^, Derar Jaradat^78,79^, Elio Jovine^80^, Chen Kuo-Hsin^81^, Rong Liu^82^, Ravi Marudanayagam^49^, Federico Mocchegiani^51^, Fabrizio Panaro^83^, Antonio Pinna^84^, Nazario Portolani^85^, Omar Saleh^86^, Claudio Sallemi^87^, Alejandro Serrablo^88^, Sameer Smadi^89^, Amal Suhool^90^, Mark A. Taylor^91^, Thomas M. van Gulik^2,3^, Ajith K. Siriwardena^92^*^#^*, Horacio J. Asbun^93^*^#^*

**Shared first author
^#^ Shared last author*

Author affiliation:

^1^Department of Surgery, Poliambulanza Foundation Hospital, Brescia, Italy
^2^Department of Surgery, University Hospital Southampton NHS Foundation Trust, Southampton, United Kingdom
^3^Department of Surgery, Amsterdam UMC location University of Amsterdam, Amsterdam, The Netherlands
^4^Cancer Centre Amsterdam, Amsterdam, The Netherlands
^5^The Intervention Centre and Department of HPB surgery, Oslo University Hospital and Institute of Medicine, University of Oslo, Oslo, Norway
^6^Department of Digestive Minimally Invasive Surgery, Antoine Béclère Hospital, Paris, France
^7^Department of Digestive and Hepatobiliary/Pancreatic Surgery, Groeninge Hospital, Kortrijk, Belgium
^8^Department of General Surgery, Centro hospitalar unversitario de Lisboa Central, Lisbon, Portugal
^9^Division of General and Transplant Surgery, University of Pisa, Pisa, Italy
^10^Digestive Health Institute, AdventHealth Tampa, Tampa, Florida, United States of America
^11^Department of General and Oncological Surgery, Umberto I Mauriziano Hospital, Turin, Italy
^12^Department of Surgery, Onze Lieve Vrouwe Gasthuis, Amsterdam, The Netherlands
^13^Department of Clinical Medicine and Surgery, Federico II University Naples, Naples, Italy
^14^Department of General, Visceral, Vascular and Transplant Surgery, University Hospital Magdeburg, Magdeburg, Germany
^15^Hepatobiliary Surgery Division, IRCCS San Raffaele Hospital, Milan, Italy
^16^Faculty of Medicine, Vita-Salute San Raffaele University, Milan, Italy
^17^Hepatobiliary Centre, Paul Brousse Hospital - Paris Sud University, Villejuif, France
^18^Department of Surgery, Virginia Mason Medical Centre, Seattle, Washington, United States of America
^19^Department of Surgery, University of California San Francisco, San Francisco, California, United States of America
^20^HPB and Liver Transplantation Unit, Department of Surgery, University Clinic, Universidad de Navarra, Pamplona, Navarra, Spain
^21^Institute of Health Research of Navarra (IdisNA), Pamplona, Spain
^22^Department of digestive, HBP and Liver Transplantation Surgery, Pitié-Salpêtrière Hospital, AP-HP, Paris, France
^23^Department of Surgery, Salmanyia Medical Complex, Manama, Bahrain
^24^Division of General Surgery and Liver Transplantation, S. Camillo Forlanini Hospital, Rome, Italy
^25^Department of Surgical, Oncological and Gastroenterological Sciences, General Surgery 2, Hepatopancreatobiliary Surgery and Liver Transplantation, Padua University Hospital, Padua, Italy
^26^Unit of Hepatobiliary Surgery and Liver Transplantation, University Hospital Reina Sofia, Cordoba, Spain
^27^Department of Surgery, Hospital Universitari Germans Trias I Pujol, Barcelona, Spain
^28^Department of Surgery, Hospital Mutua de Terrassa, Barcelona, Spain
^29^Hepato-Pancreato-Biliary Surgery and Liver Transplantation Unit, University of Modena and Reggio Emilia, Modena, Italy
^30^Department of Surgery, Cambridge University Hospitals NHS Foundation Trust, Cambridge, United Kingdom
^31^Liver Unit, Department of General Surgery, Univeristy Hospital of Wales, Cardiff, United Kingdom
^32^Department of Hepato-Pancreato-Biliary Surgery, Moscow Clinical Scientific Centre, Moscow, Russia
^33^Department of General and Digestive Surgery, Hospital Clinic, Barcelona, Spain
^34^The Intervention Centre, Oslo University Hospital, Oslo, Norway
^35^Department of Surgery, Fondazione Policlinico Universitario A. Gemelli IRCCS, Rome, Italy
^36^Department of Surgery, Universitair Medisch Centrum Utrecht, Utrecht, The Netherlands
^37^Department of Surgery, Seoul National University Bundang Hospital, Seoul National University College of Medicine, Seoul, South-Korea
^38^Department of Surgery, Institute of Gastroenterology, Tokyo Women's Medical University, Tokyo, Japan
^39^Department of HPB surgery and liver transplantation, AP-HP, Beaujon Hospital, DMU DIGEST, Clichy, France
^40^Department of Surgery, Leeds Teaching Hospitals NHS Trust, Leeds, United Kingdom
^41^Servei de Cirurgia General i Digestiva, Hospital Doctor Josep Trueta de Girona, Girona, Catalonia, Spain
^42^Department of Hepato-Pancreatc-Biliary Surgery, “F. Miulli” General Regional Hospital, Acquaviva delle Fonti, Bari, Italy
^43^Department of Medicine and Surgery, LUM University, Casamassima, Bari, Italy
^44^Department of Transplantation and Hepatopancreatobiliary Surgery, King's College Hospital NHS Foundation Trust, London, United Kingdom
^45^Department of General and Hepatopancreatobiliary Surgery, Ghent University Hospital, Ghent, Belgium
^46^Department of General, Visceral and Transplantation Surgery, Clinic and University Hospital Virgen de la Arrixaca, IMIB-ARRIXACA, El Palmar, Murcia, Spain
^47^Department of Surgery, University of Verona, Verona, Italy
^48^Department of General and Digestive Surgery, Vithas Hospital Malaga, Malaga, Spain
^49^Department of General, Visceral and Transplant Surgery, Hannover Medical School, Hannover, Germany
^50^Department of Oncologic Surgery, Regional Cancer Institute, Montpellier, France
^51^Liver Unit, Queen Elizabeth Hospital, Birmingham, United Kingdom
^52^Hepatobiliary Surgery section, Makati Medical Centre, Makati, The Philippines
^53^HPB and Abdominal Transplantation Surgery, Department of experimental and clinical medicine, University Hospital of Marche, Polytechnic University of Marche, Ancona, Italy
^54^Department of Surgery, Ageo Central General Hospital, Ageo, Japan
^55^Department of Surgery, Newcastle upon Tyne Hospitals NHS Foundation Trust, Newcastle upon Tyne, United Kingdom
^56^PLANETS Cancer Charity, Emsworth, Hampshire, United Kingdom
^57^Department of Surgical Science, HPB, and Transplant Unit, University of Rome Tor Vergata, Rome, Italy
^58^Hepato Biliary Pancreatic (HPB) Surgery Unit, Pederzoli Hospital, Peschiera del Garda, Italy
^59^Department of General Surgery, Division of Hepatopancreatobiliary Surgery, Koc University Hospital, Istanbul, Turkey
^60^Hepatobiliary Surgery Unit, Fondazione Policlinico Universitario A. Gemelli, IRCCS, Catholic University of the Sacred Heart, Rome, Italy
^61^Department of Visceral Surgery, University Hospital Basel, Basel, Switzerland
^62^Department of General and Digestive Surgery, Corachan Clinic, Barcelona, Spain
^63^department of Visceral, Transplant and Thoracic Surgery, Medical University Innsbruck, Innsbruck, Austria
^64^Department of Surgery, MASHHAD University of Medical Sciences, Mashhad, Iran
^65^Department of Hepatobiliary and General Surgery, Royal Liverpool University Hospital, Liverpool, United Kingdom
^66^(No affiliation data provided)
^67^Department of General Surgery, University Hospitals Coventry and Warwickshire, Clifford Bridges Road, Coventry, United Kingdom
^68^Pancreato-Biliary and Robotic Surgery, HPB and Transplant Surgeon, Hospital Clinic, Barcelona, Spain
^69^Department of Clinical and Experimental Sciences, University of Brescia, Brescia, Italy
^70^Department of General and Oncological Surgery, Ferrero Hospital, Verduno, italy
^71^Department of Surgery and Transplantation, Niguarda Hospital Milan & University of Milano-Bicocca School of Medicine and Surgery, Milan, Italy
^72^Department of Surgery, AGIA OLGA Hospital, Athens, Greece
^73^Unit of Statistics, Istituto Ospedaliero Fondazione Poliambulanza, Brescia, Italy
^74^HPB & Liver Transplant Unit, Royal Free London, London, United Kingdom
^75^Department of Surgery, Division of Hepatobiliary and Pancreatic Surgery, University of Pittsburgh Medical Centre, Pittsburgh, Pennsylvania, United States of America
^76^Department of Hepatopancreatobiliary and Transplant Surgery, Singapore General Hospital and National Cancer Centre Singapore, Singapore, Singapore
^77^Surgery Academic Clinical Programme, Duke-National University of Singapore Medical School, Singapore, Singapore
^78^HPB Surgery, Atrium Health, Charlotte, North Carolina, United States of America
^79^Department of Surgery, Medical University of Graz, Graz, Austria
^80^Department of Surgery, University Hospital Essen, Essen, Germany
^81^Department of Surgery, IRCCS Azienda Ospedaliero-Universitaria di Bologna, Bologna, Italy
^82^Department of Surgery, Far-Eastern Memorial Hospital, New Taipei City, Taiwan
^83^Faculty of Hepatopancreatobiliary Surgery, The First Medical Centre of Chinese People’s Liberation Army (PLA) General Hospital, Beijing, China
^84^Department of Surgery, Montpellier University Hospital, Montpellier, France
^85^Department of Abdominal and Transplant Surgery, Cleveland Clinic Florida, Weston, Florida, United States of America
^86^Department of Clinical and Experimental Sciences, Surgical Clinic, University of Brescia, Brescia, Italy
^87^Liver and Pancreatic Cancer Charity
^88^Department of Intervention Radiology, Fondazione Poliambulanza Istituto Ospedaliero, Brescia, Italy
^89^HPB Surgical Division, Miguel Servet University Hospital, Zaragoza, Spain
^90^Department of Surgery, King Hussein Medical Centre, Amman, Jordan
^91^Department of Hepatopancreaticobiliary and Endocrine Surgery, Sheikh Shakhbout Medical City (SSMC), Abu Dhabi, United Arab Emirates
^92^Department of Surgery, Mater Hospital, Belfast, Northern Ireland, United Kingdom
^93^Hepatobiliary and Pancreatic Surgery Unit, Manchester University NHS FT, Manchester, United Kingdom
^94^Hepato-Biliary and Pancreas Surgery, Miami Cancer Institute, Miami, Florida, United States of America

**Corresponding author:** Prof. Mohammad Abu Hilal, The University of Jordan, School of Medicine, Queen Rania St, Amman, Jordan, [abuhilal9@gmail.com](mailto:abuhilal9@gmail.com), ORCIDid: 0000-0002-3162-4639; Twitter: @abuhilal9abu

**Supplementary Materials - Index**

| **Supplementary Appendixes** |  |
| --- | --- |
| Appendix 1. Panels and committees | *pag. 5* |
| Appendix 2. MILS search terms | *pag. 8* |
| Appendix 3. GRADE scoring system | *pag. 9* |
| Appendix 4. Summary of Findings Tables and Considered Judgement | *pag. 10* |
| Appendix 5. Strength of Recommendation | *pag. 13* |
| Appendix 6. Professional Oaths | *pag. 14* |
| Appendix 7. AGREE II GRS Tool | *pag. 15* |
| Appendix 8. Jury Feedback Form | *pag. 16* |
| **Supplementary Figures and Tables** |  |
| Supplementary Table 1. Domain 1 MILS Terminology | *pag. 19* |
| Supplementary Table 2. Domain 2 Clinical Questions and Recommendations | *pag. 22* |
| Supplementary Table 3. Domain 3 Clinical Questions and Recommendations | *pag. 25* |
| Supplementary Table 4. Domain 4 Clinical Questions and Recommendations | *pag. 28* |
| Supplementary Table 5. Domain 5 Clinical Questions and Recommendations | *pag. 31* |
| Supplementary Table 6. Domain 6 Clinical Questions and Recommendations | *pag. 35* |
| Supplementary Table 7. Domain 7 Clinical Questions and Recommendations | *pag. 37* |
| Supplementary Table 8. Domain 8 Clinical Questions and Recommendations | *pag. 42* |
| Supplementary Table 9. Agreement Rates and Topic Mean Performance Scores | *pag. 51* |

**Supplementary Appendixes**

Appendix 1. Panels and committees

| **Steering Committee** |
| --- |
| Prof. Mo Abu Hilal |
| Prof. Luca Aldrighetti |
| Dr. Rutger-Jan Swijnenburg |
| Prof. Mathieu D’Hondt |
| Prof. Daniel Cherqui |
| Prof. H.P. Marques |
| Prof. Fernando Rotellar |
| Prof. B. Edwin |
| Prof. I. Dagher |

| **Domain 1** | **Domain 2** | **Domain 3** | **Domain 4** |
| --- | --- | --- | --- |
| Prof. Adnan Alseidi (chair) | Prof. Fernando Rottelar (chair) | Prof. Mathieu D'Hondt (chair) | Dr. Rutger-Jan Swijnenburg (chair) |
| Prof. Ugo Boggi (chair) | Prof. Iswanto Sucandy (chair) | Prof. Ibrahim Dagher (chair) | Prof. Alessandro Ferrero (chair) |
| Prof. Ho-Seong Han | Prof. Go Wakabayashi | Prof. Constantino Fondevilla | Prof. Fabrizio Di Benedetto |
| Dr. Hendrik Marsman | Prof. Luca Aldrighetti | Prof. Krishna Menon | Prof. Esteban Cugat |
| Prof. Santiago Lopez Ben | Prof. Mikhail Efanov | Prof. Mikhail Efanov | Prof. Steven White |
| Prof. Moritz Schmelzle | Prof. Peter Lodge | Prof. Riccardo Robles-Campos | Dr. Hani Alsaati |
| Prof. Alessandro Ferrero | Prof. Umberto Cillo | Prof. Giuseppe Ettorre | Prof. Roland Croner |
| Dr. Federica Cipriani | Prof. Daniel Cherqui | Dr. Ruben Ciria | Prof. Santiago Lopez Ben |
| Dr. Miriam Cortes | Dr. Giammauro Berardi | Dr. Trish Duncan | Prof. Andrea Ruzzenente |
| Dr. Simone Famularo (lead) | Dr. Francesca Ratti | Prof. Felice Giuliante | Dr. Rafael Diaz-Nieto |
| Dr. Sara Saidi | Soufyan El Adel | Dr. Tijs Hoogteijling (lead) | Dr. Jasper Sijberden (lead) |
| Dr. Victor Lopez-Lopez | Dr. Victor Lopez-Lopez (lead) | Dr. Andrea Sheel | Dr. Fernando Pardo Aranda |
| Dr. Emre Bozkurt (co-lead) | Dr. Maria Conticchio | Dr. Fancesco Taliente | Dr. Roberta Angelico |
| Dr. Roberta Angelico | Dr. Florian Primavesi | Dr. Andrea Benedetti Cacciaguerra (co-lead) | Dr. Christoph Kuemmerli (co-lead) |
|  | Dr. Elisa Bannone | Dr. Simone Famularo (lead) | Dr. Andrea Sheel |
|  |  | Dr. Martina Guerra |  |
|  |  |  |  |

| **Domain 5** | **Domain 6** | **Domain 7** | **Domain 8** |
| --- | --- | --- | --- |
| Prof. Bjorn Edwin (chair) | Prof. Hugo Marques (chair) | Prof. Adnan Alseidi (chair) | Prof. Andrew Gumbs (chair) |
| Dr. Hendrik Marsman (chair) | Prof. Olivier Scatton (chair) | Prof. Roberto Troisi (chair) | Prof. Roland Croner (chair) |
| Dr. Asmund Fretland | Prof. Steven White | Dr. Rutger-Jan Swijnenburg | Dr. Asmund Fretland |
| Prof. Jeroen Hagendoorn | Prof. Marco Vivarelli | Prof. Mikhael Lesurtel | Prof. Iswanto Sucandy |
| Prof. Robert Sutcliffe | Prof. Mikhael Lesurtel | Prof. Umberto Cillo | Dr. Niki Rashidian |
| Prof. Roberto Troisi | Dr. Catherine Teh | Prof. Robert Sutcliffe | Prof. Bjorn Edwin |
| Prof. Julio Santoyo | Dr. Nadia Russolillo | Dr. Federica Cipriani | Dr. Emre Bozkurt (lead) |
| Prof. Riccardo Memeo | Dr. Christoph Kuemmerli (lead) | Dr. Niki Rashidian | Dr. Niki Rashidian (co-lead) |
| Prof. Goro Honda | Dr. Jasper Sijberden (co-lead) | Dr. Florian Primavesi (lead) | Dr. Martina Guerra |
| Prof. Mortiz Schmelzle | Soufyan El Adel | Dr. Niki Rashidian (co-lead) | Dr. Elisa Bannone |
| Dr. Ruben Ciria | Dr. Fernando Pardo Aranda | Dr. Gabriela Pilz da Cunha | Teodros Veronesi |
| Dr. Olivia Sgarbura | Teodros Veronesi | Dr. Maria Conticchio | Dr. Sara Saidi |
| Dr. Andrea Benedetti Cacciaguerra (lead) |  | Dr. Francesco Sucameli |  |
| Dr. Gabriela Pilz da Cunha |  |  |  |
| Dr. Francesco Sucameli |  |  |  |
| Dr. Tijs Hoogteijling (co-lead) |  |  |  |
| Dr. Fancesco Taliente |  |  |  |

| **Validation Committee** |
| --- |
| Prof. Ajith Siriwardena |
| Prof. M.G.H. Besselink |
| Prof. Christos Dervenis |
| Dr. Alejandro Serrablo |
| Dr. Mark Taylor |
| Prof. Brian Goh |
| Prof. Horacio Asbun |
| Prof. David Geller |
| Prof. Alfredo Guglielmi |
| Prof. Luciano De Carlis |
| Prof. Sergio Alfieri |
| Prof. Elio Jovine |
| Prof. Antonio Pinna |
| Dr. Chen Kiu-Hsin |
| Dr. Nazzario Portolani |
| Dr. Claudio Sallemi |
| Dr. Gianluca Baiocchi |
| Prof. Safi Dokmak |
| Dr. Sameer Smadi |
| Dr. Federico Mocchegiani |
| Dr. Fabio Ausania |
| Prof. Kito Fusai |
| Dr. Amal Suhool |
| Prof. Ravi Marudanayagam |
| Dr. Antonella Delvecchio |
| Mr. Omar Saleh |
| Prof. Jawad Ahmad |
| Prof. Thomas van Gulik |
| Dr. Fabrizio Panaro |
| Dr. Stefano Berti |
| Ms. Clarissa Ferrari |
| Prof. Rong Liu |
| Dr. Derar Jaradat |
| Dr. David Iannitti |

| **Jury Committee** |
| --- |
| prof. Brian Goh |
| Prof. Alfredo Guglielmi |
| Prof. Elio Jovine |
| Dr. Fabio Ausania |
| Prof. Kito Fusai |
| Prof. Thomas van Gulik |
| Dr. Fabrizio Panaro |
| Dr. Derar Jaradat |

Appendix 2. MILS search terms

("Hepatectomy"[Mesh] OR hepatectom*[tiab] OR ((liver[tiab] OR hepato*[tiab] OR hepatic*[tiab]) AND (surger*[tiab] OR resect*[tiab] OR operation[tiab] OR procedure*[tiab]))) AND ("Robotic Surgical Procedures"[Mesh] OR robot*[tiab] OR "Video-Assisted Surgery"[Mesh] OR video-assist*[tiab] OR "Laparoscopy"[Mesh] OR laparoscop*[tiab] OR "Minimally Invasive Surgical Procedures"[Mesh:NoExp] OR minimal-invasiv*[tiab] OR minimally-invasiv*[tiab] OR mini-invasive[tiab] OR minimal-access[tiab] OR keyhole[tiab] OR less-invasiv*[tiab])

Appendix 3. GRADE scoring system


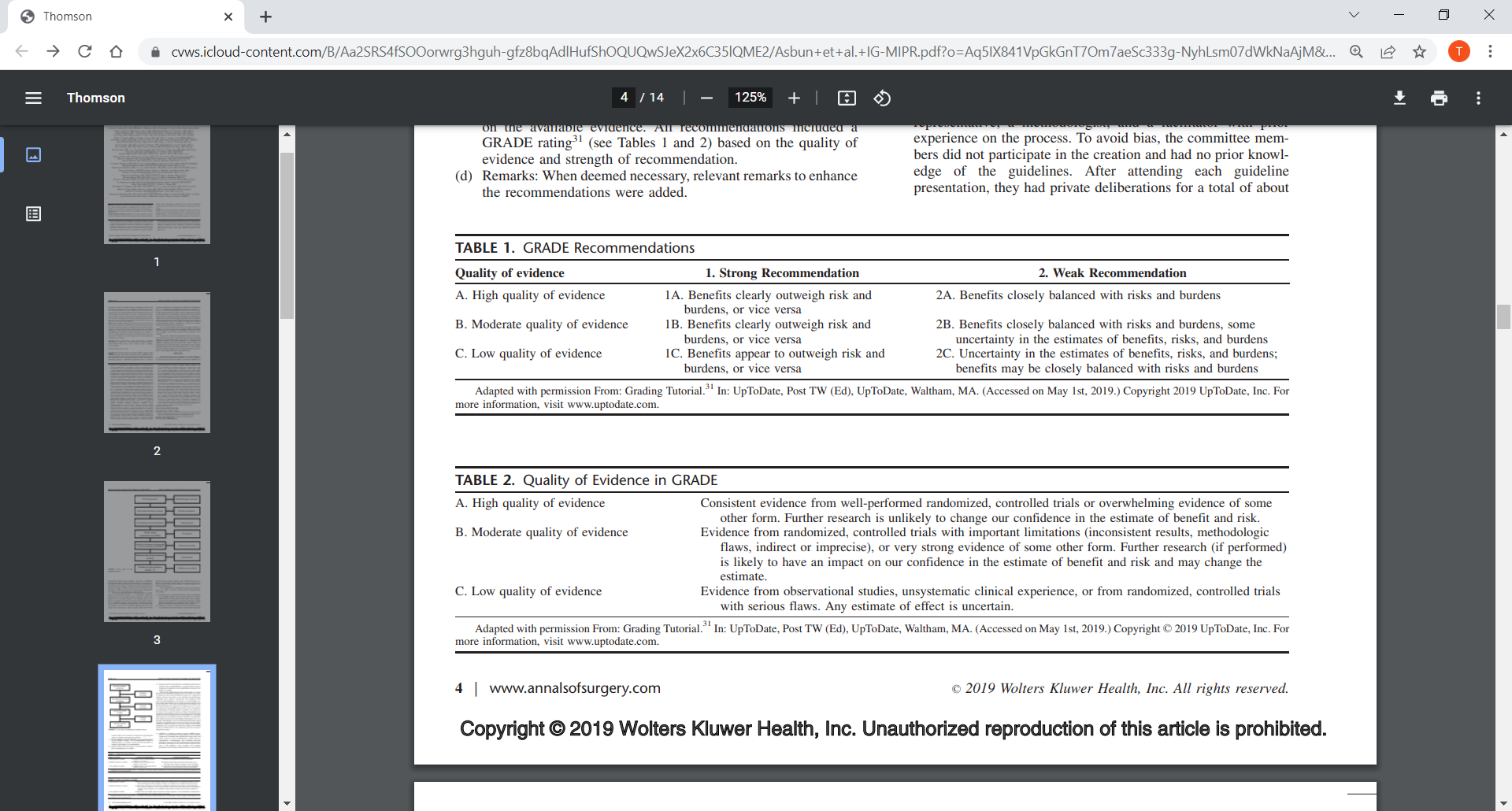


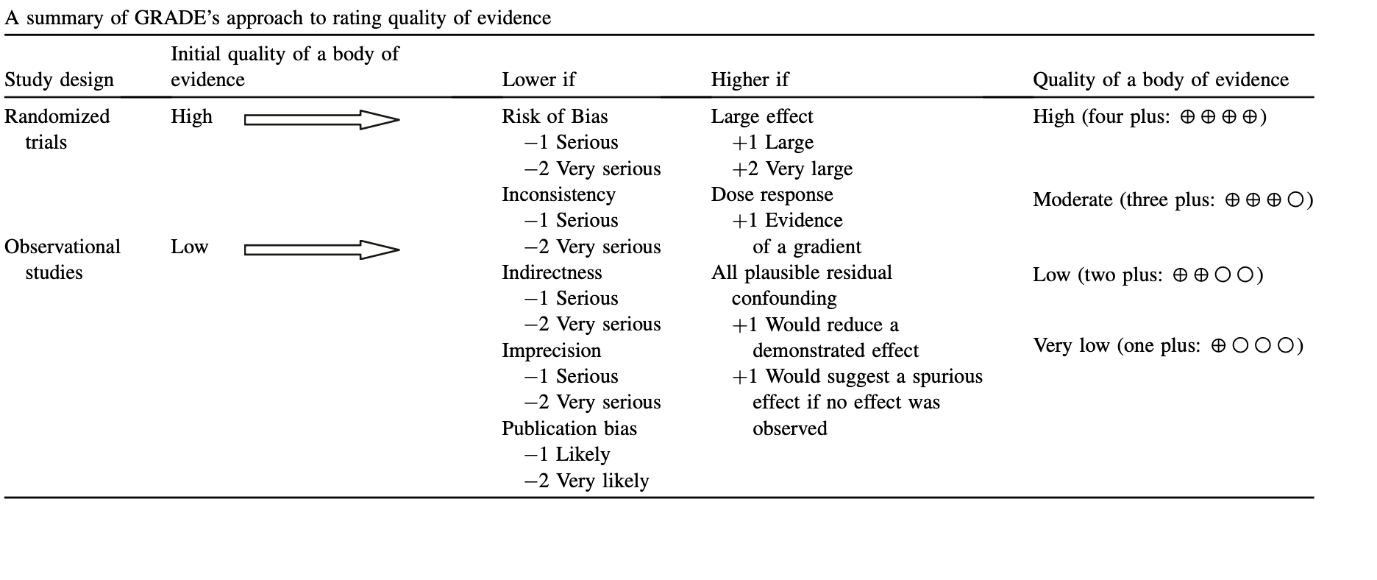


Appendix 4. Summary of Findings Tables and Considered Judgement


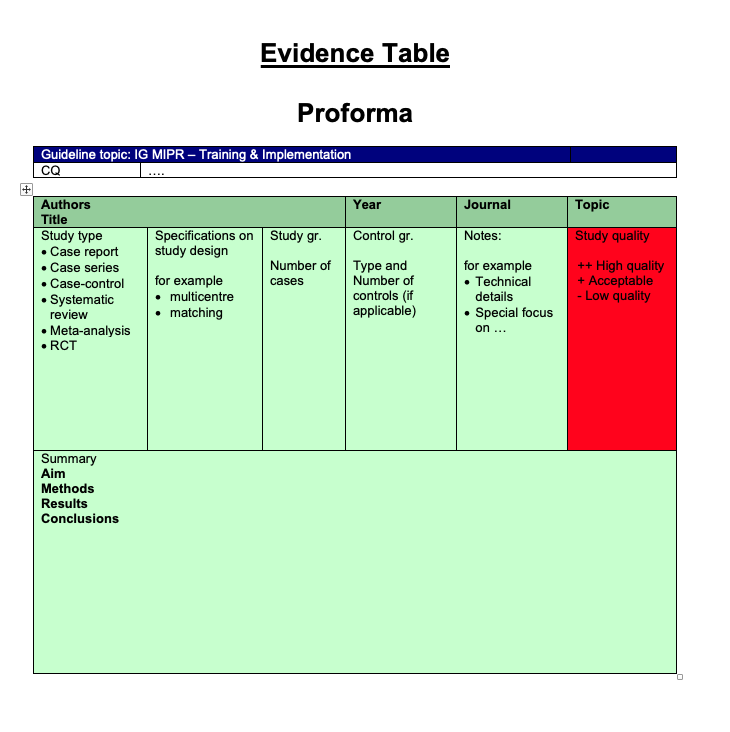


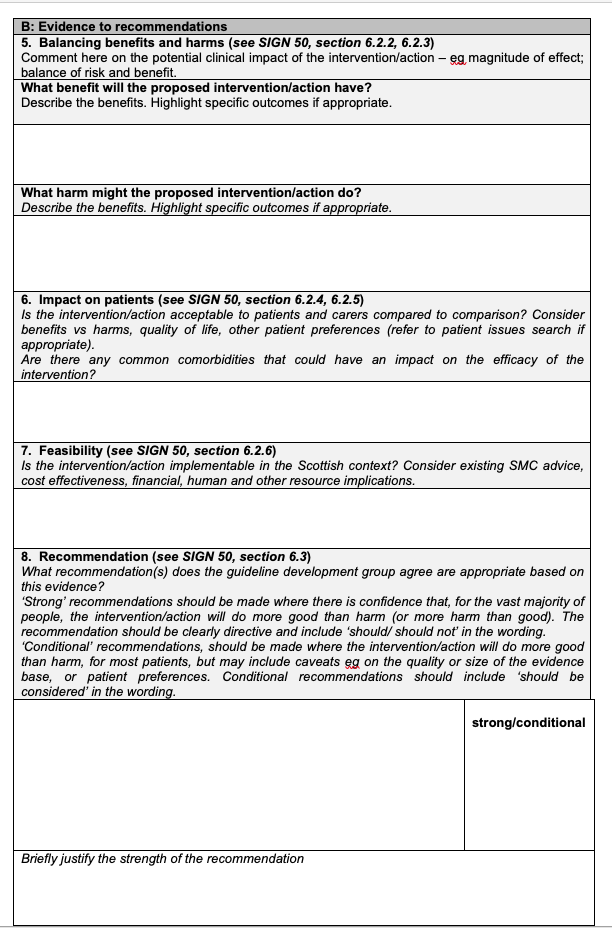

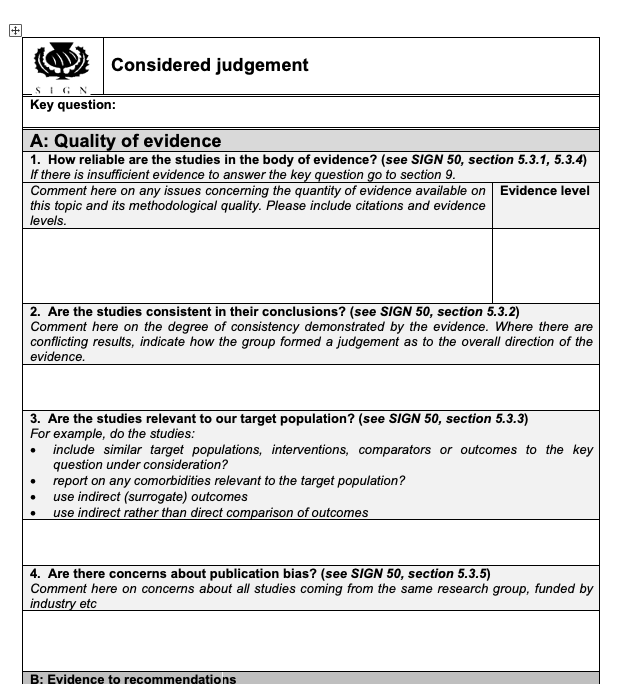


Appendix 5. Strength of Recommendation


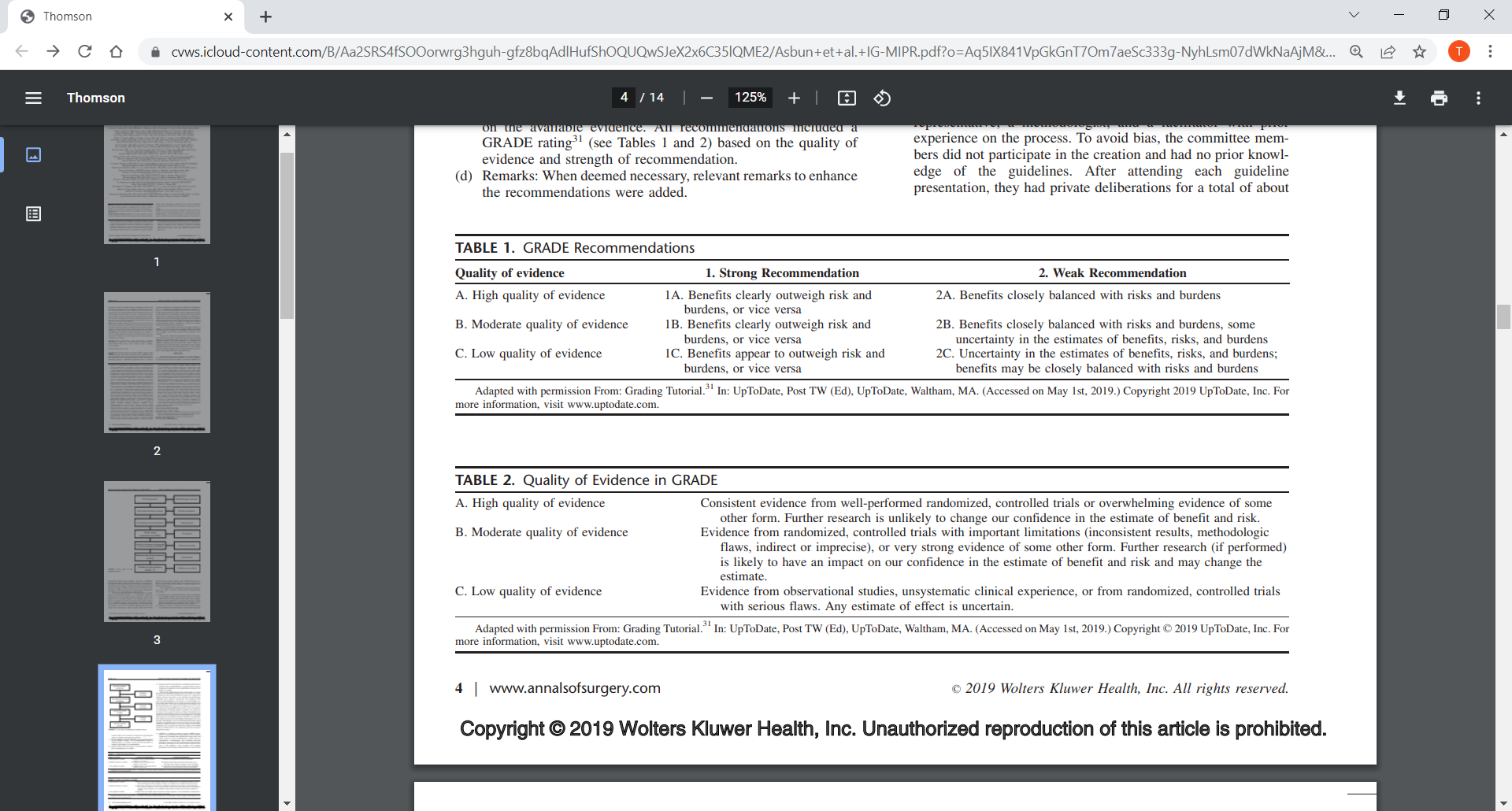

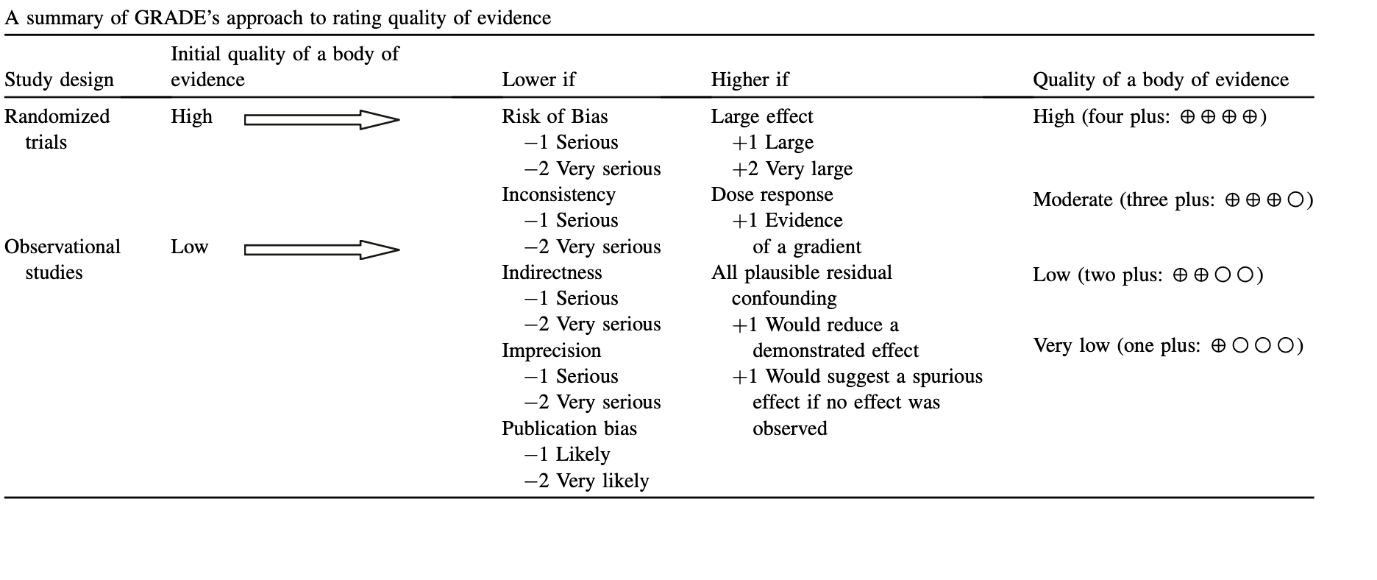


Appendix 6. Professional Oaths

Expert Committee – Björn Edwin

I, Björn Edwin, on behalf of the Expert Committee, declare that we have operated following the norms of professional and ethical integrity and acted to our best scientific knowledge, ability, and judgment in accordance with the internationally acknowledged methodology for guidelines development. We will work with the Chairman, the Validation, the Research, and the Jury Committee to finalize the First Internationally Validated Guidelines on Minimally Invasive Liver Surgery with no conflict of interest to report.

Validation Committee – Horacio Asbun

I, Horacio Asbun, on behalf of the Validation Committee, declare that we have regularly been informed about the ongoing process of guideline development by the chairman professor Mohammad Abu Hilal and that we will validate what is presented by the Expert and Research Committee in accordance with the AGREE-methodology. We will operate in accordance with the norms of professional and ethical integrity, and act to our best scientific knowledge, ability, and judgment.

Research Committee – Tijs Hoogteijling

I, Tijs Hoogteijling, on behalf of the Research Committee, declare that we have operated following the norms of professional and ethical integrity and acted to our best scientific knowledge, ability, and judgment in accordance with the internationally acknowledged methodology for guidelines development. We will work with the Chairman, the Expert, the Validation, and the Jury Committee to finalize the First Internationally Validated Guidelines on Minimally Invasive Liver Surgery with no conflict of interest to report.

Appendix 7. AGREE II GRS Tool


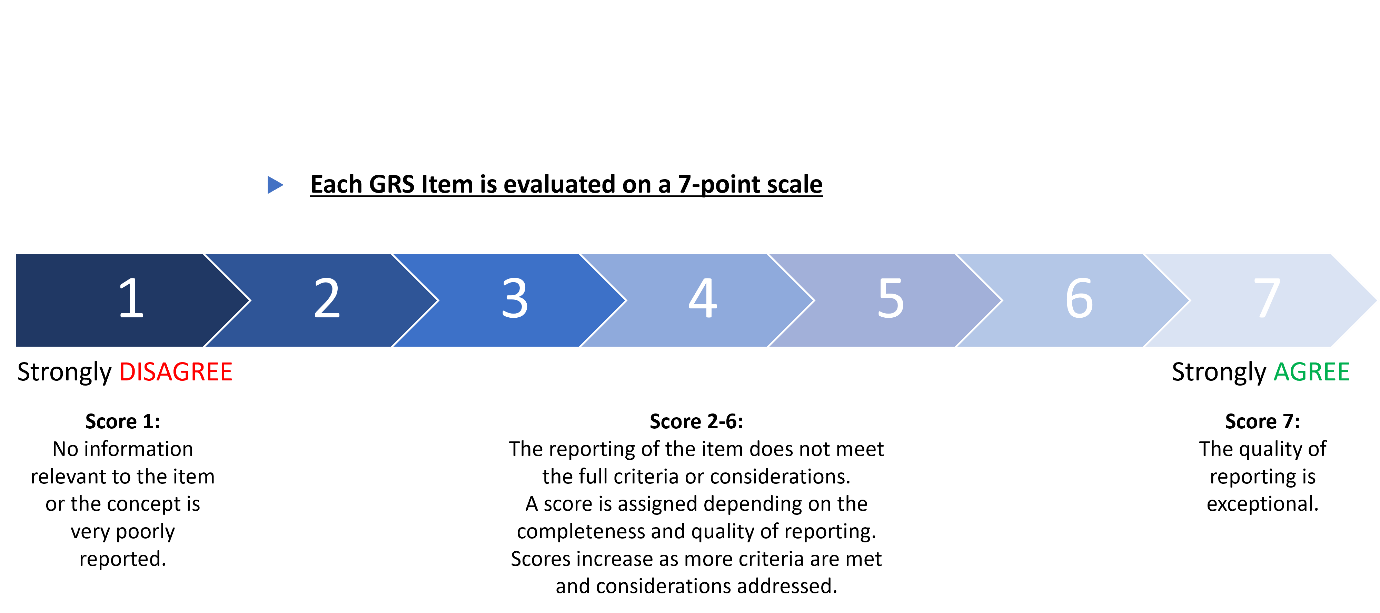


GRS Items:


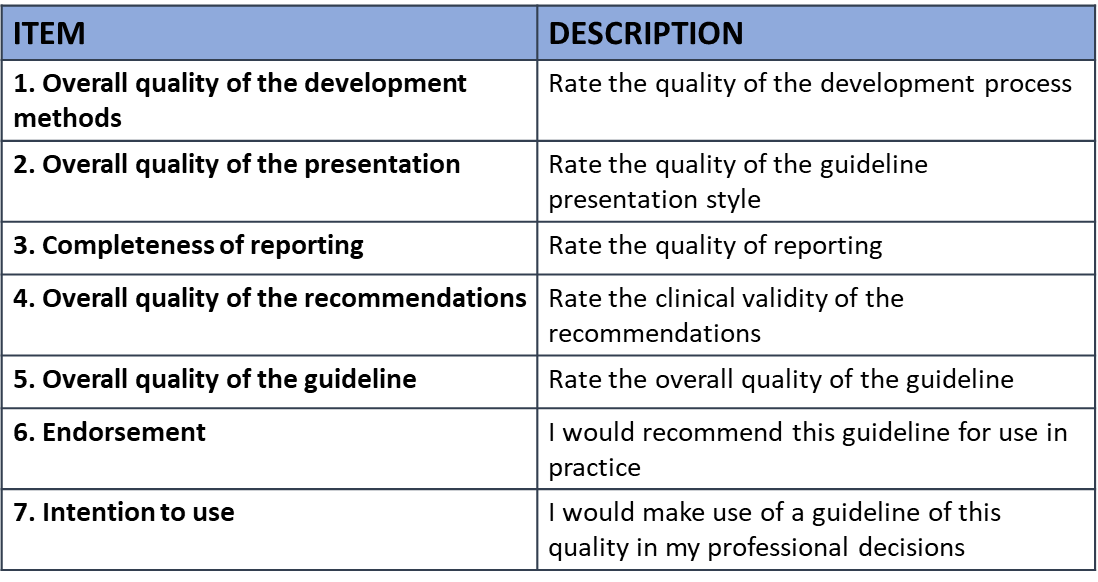


Example of mean performance scores:


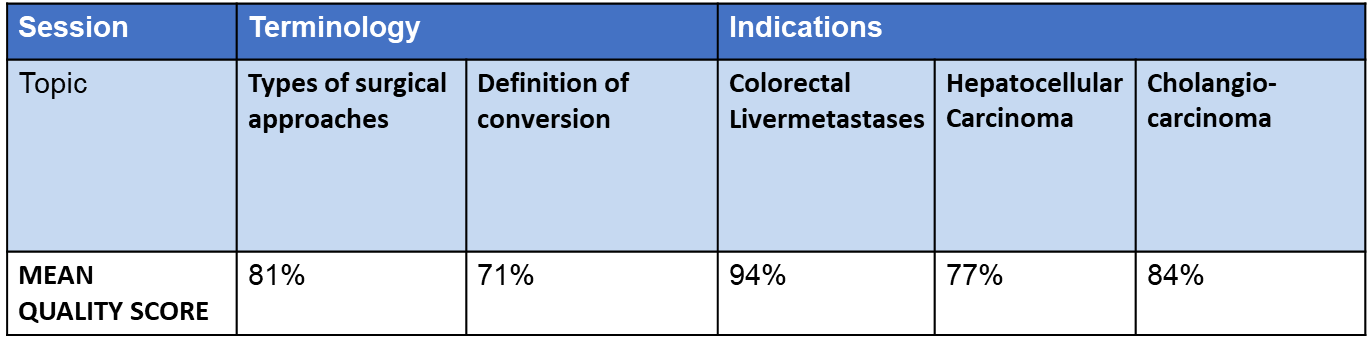


Appendix 8. Jury Feedback Form

**JURY COMMITTEE FEEDBACK FORM**

FEEDBACK FORM - DAY 1

NAME/SURNAME:

 ____________________________________

| **Questions** | Lowest grade  (1) |  | Highest grade  (10) |
| --- | --- | --- | --- |
| *Methodology* | | | |
| 1. The methodology was all clear to the public. | 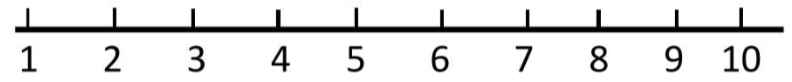 | | |
| *Committee / public involvement* | | | |
| 1. The validation committee was present on every assessment. | 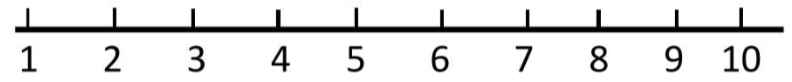 | | |
| 1. The validation committee had enough privacy during the validation process. | 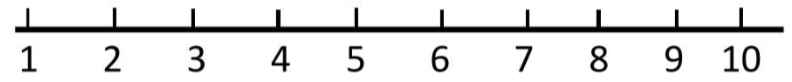 | | |
| 1. The public had an independent vote on each statement. | 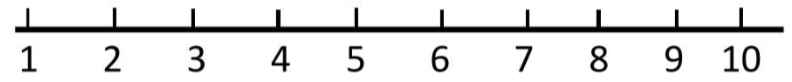 | | |
| 1. The patients’ representative involvement was adequate. | 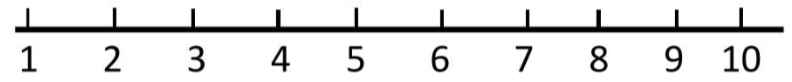 | | |
| 1. Faculty participation level | 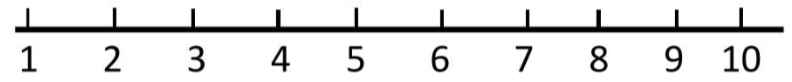 | | |
| *IEGUMILS meeting* | | | |
| 1. How would you rate the scientific level of today | 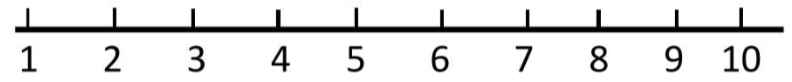 | | |
| 1. How would you rate the presentations of today | 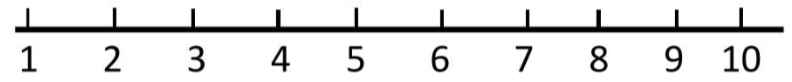 | | |
| 1. How would you rate the scientific program of today | 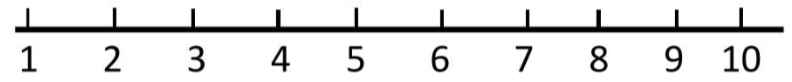 | | |
| 1. How would you rate the clinical impact of today | 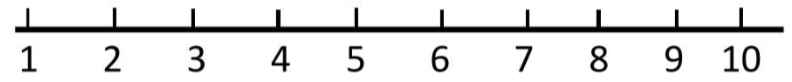 | | |
| 1. There was enough time for discussion after each statement | 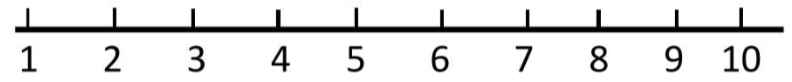 | | |
| 1. The discussions before/after the vote were democratic and not influential | 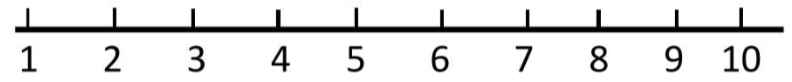 | | |
|  |  | | |

FEEDBACK FORM - DAY 2

NAME/SURNAME:

 ____________________________________

| **Questions** | Lowest grade  (1) |  | Highest grade  (10) |
| --- | --- | --- | --- |
| *Methodology* | | | |
| 1. The methodology was all clear to the public. | 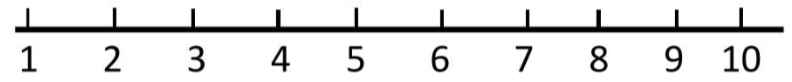 | | |
| *Committee / public involvement* | | | |
| 1. The validation committee was present on every assessment. | 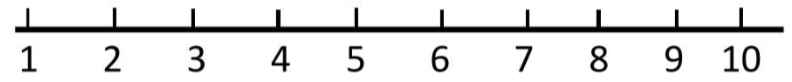 | | |
| 1. The validation committee had enough privacy during the validation process. | 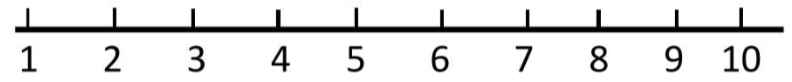 | | |
| 1. The public had an independent vote on each statement. | 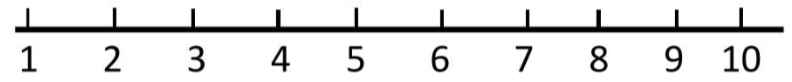 | | |
| 1. The patients’ representative involvement was adequate. | 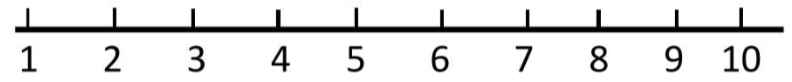 | | |
| 1. Faculty participation level | 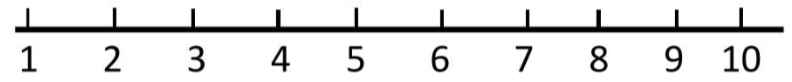 | | |
| *IEGUMILS meeting* | | | |
| 1. How would you rate the scientific level of today | 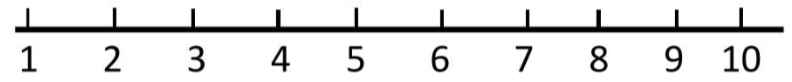 | | |
| 1. How would you rate the presentations of today | 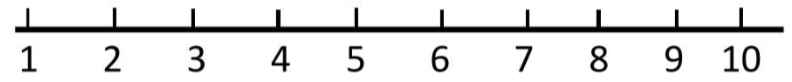 | | |
| 1. How would you rate the scientific program of today | 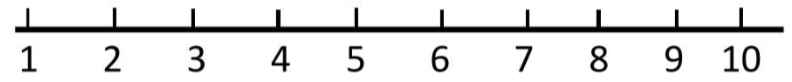 | | |
| 1. How would you rate the clinical impact of today | 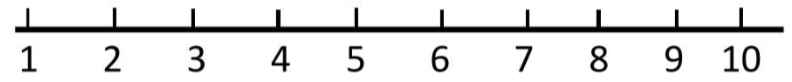 | | |
| 1. There was enough time for discussion after each statement | 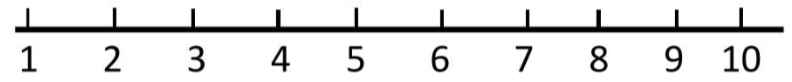 | | |
| 1. The discussions before/after the vote were democratic and not influential | 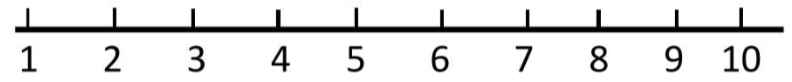 | | |

**Supplementary Figures and Tables**

Supplementary Table 1. Domain 1 MILS Terminology

| **Q** | **Clinical Question** | **R** | **Recommendation** | **Code** | **Comment** |
| --- | --- | --- | --- | --- | --- |
| 1 | Are the laparoscopic, the robotic and the open approach the only defined approaches that should be considered in data collection, registries and research? | 1 | Besides the laparoscopic, robotic, and open approaches, also robot-assisted, hand-assisted, single incision and combined approaches should be reported in surgical series as defined in table 9. | 1C | Comments added to table 9. |
| 2 | How should we define a procedure in which both the laparoscopic and robotic approaches were adopted? | 2 | If the assistant performs a significant part of the surgery laparoscopically, beyond simply assisting the surgeon on the robotic console, it should be called ‘COMBINED ROBOTIC LAPAROSCOPIC SURGERY (CRLS)’. | 1C |  |
| 3 | How should we define a procedure in which the open and laparoscopic/robotic approach were adopted? | 3 | The combination of laparoscopic/robotic and open approaches should be defined: COMBINED LAPAROSCOPIC/ROBOTIC and OPEN SURGERY (CLOS/CROS). This is a combined laparoscopic/robotic and open procedure, including a planned mini laparotomy, that is performed for reasons different from specimen extraction. Prior studies may have referred to this as a hybrid procedure. | 1C |  |
| 4 | How should we define an approach where another approach was used to perform a small part of the procedure? | 4 | When two approaches are used to perform a procedure but one of the two is clearly predominant, the procedure should be defined according to the main approach while applying the specific definitions provided by these guidelines. | 1C |  |
| 5 | What is the role of the transthoracic and/or transdiaphragmatic approach in MILS? | 5 | The transthoracic route can be considered in patients with a history of multiple upper abdominal surgeries, or in posterosuperior resections. It allows only limited hepatic resections. The superiority of this approach versus an abdominal approach has not been demonstrated. | 2C |  |
| 6 | How should we define minor hepatectomies in the posterosuperior segments? | 6 | When performed laparoscopically or robotically, resections in the posterosuperior segments are technically complex resections. | 1C |  |
| 7 | How should we define the passage from laparoscopic to robotic or vice versa if this was not intended? | 7 | The primary approach, which is identified on the intention-to-treat purpose, should be used in both elective and emergency situations, and disclosed accordingly when switching from laparoscopic to robotic or vice versa. | 1C |  |
| 8 | Do all conversions in laparoscopic liver resection (LLR) and robotic liver resection (RLR) have the same impact on patient’s outcome? | 8 | Emergency conversions in LLR or RLR are associated with worse outcomes. Conversion to open surgery should ideally be pre-emptive when surgical difficulties are anticipated. | 1B |  |
| 9 | How should we define an elective conversion in LLR and RLR? | 9 | An elective conversion in LLR or RLR occurs in hemodynamically stable patients because of unanticipated conditions (e.g. concerns about resection margins, failure to progress, technology failure, etc) that require a switch to a different surgical approach. | 1B |  |
| 10 | How should we define an emergency conversion in LLR and RLR? | 10 | In LLR and RLR, "emergency conversion" refers to a necessary decision required to fix potentially life-threatening conditions or serious intraoperative complications, that cannot be properly handled while maintaining the laparoscopic or robotic approach. | 1B | Unplanned, Emergent, urgent, and reactive are synonyms that were used interchangeably in prior literature. |
| 11 | How should we define an unintended conversion in LLR and RLR? | 11 | In LLR and RLR, the unplanned (emergent or non-emergent) use of a laparotomy to complete the procedure should be classified as unintended conversion. However, planned (elective or proactive) conversion would fall under ‘Combined Laparoscopic-Open Surgery’ or “combined robotic-open surgery” as defined above. | 1B |  |

Supplementary Table 2. Domain 2 Clinical Questions and Recommendations

| **Q** | **Clinical Question** | **R** | **Recommendation** | **Code** |
| --- | --- | --- | --- | --- |
| 12 | What is the role of laparoscopic liver resection (LLR) in the management of CRLM? | 12 | LLR should be considered as a valid alternative approach to open surgery in the management of CRLM. It seems to be associated with improved short-term and at least similar long-term outcomes compared with open surgery in patients with CRLM, when performed in centres with adequate experience and expertise. | 1A |
| 13 | What is the role of robotic liver resection (RLR) in the management of CRLM? | 13 | RLR offers similar short- and long-term results to LLR in the management of CRLM when performed in centres with adequate experience and expertise. | 2B |
| 14 | What is the role of LLR in the management of synchronous CRLM when simultaneous resection is indicated? | 14 | When simultaneous resection is indicated for synchronous CRLM the laparoscopic approach should be considered as an alternative to open surgery. It seems to be associated with improved short-term and with similar long-term outcomes compared with open surgery when performed in centres with adequate experience and expertise. | 2B |
| 15 | What is the role of RLR in the management of synchronous CRLM when simultaneous resection is indicated? | 15,1 | RLR is a safe and feasible alternative option for simultaneous resection of colon cancer and synchronous CRLM for selected patients in experienced hands. | 2C |
|  |  | 15,2 | Defining the role of RLR is important. Further studies to assess this are recommended. | 1C |
| 16 | What is the role of LLR in the management of noncolorectal liver metastases (NCRLM)? | 16 | LLR should be considered an alternative to open surgery in the management of NCRLM. It seems to be associated with improved short-term and with similar long-term outcomes compared with open surgery, when performed in centres with adequate experience and expertise. | 1B |
| 17 | What is the role of RLR in the management of NCRLM? | 17 | RLR should be considered an alternative to open surgery in the management of NCRLM. Further studies are required. | 2C |
| 18 | What is the role of LLR in the management of hepatocellular carcinoma (HCC)? | 18 | When feasible, LS should be preferred to open surgery in the management of HCC. It is associated with improved short-term and with similar long-term outcomes compared to open surgery, when performed in centres with adequate experience and expertise. | 1B |
| 19 | What is the role of RLR in the management of HCC? | 19,1 | RLR for HCC is as safe and feasible as conventional open and laparoscopic approaches, offering comparable surgical and oncological outcomes in selected patients when performed in centres with adequate experience and expertise. | 2B |
|  |  | 19,2 | Difficulty scores are crucial for proper case selection, warranting investigation in randomized controlled trials. | 1C |
| 20 | What is the role of LLR in the management of intrahepatic cholangiocarcinoma (iCCA)? | 20 | LLR should be considered a valid alternative approach to open surgery in the management of iCCA. | 2C |
| 21 | What is the role of RLR in the management of intrahepatic iCCA? | 21 | Little evidence is available in the literature regarding RLR for iCCA. Further studies are required in this area. | 2C |
| 22 | What is the role of LLR and RLR in the management of hilar CCA? | 22 | LLR and RLR have been reported for hilar cholangiocarcinoma but there are little data to recommend their routine use. | 2C |
| 23 | What is the role of LLR and RLR in the management of benign liver disease? | 23 | LLR and RLR should be considered a valid alternative approach to open surgery in the management of benign liver disease. It is associated with improved short-term outcomes compared with open surgery, when performed in centres with adequate experience and expertise. However, this should not extend or change the indications for surgery. | 1B |
| 24 | What is the role of LLR and RLR in the setting of living donor? | 24,1 | LLR and RLR for living donor major hepatectomy for adult transplantation have been reported and may be a valid alternative to open surgery, when performed in centres with adequate experience and expertise. | 1B |
|  |  | 24,2 | For paediatric transplantation, LLR should be considered as standard practice similar to the open technique, whereby pure laparoscopic left lateral sectionectomy offers a reduction in blood loss, morbidity, and hospital stay. | 1B |
| 25 | What is the role of LLR and RLR in the setting of liver resection requiring combined biliary reconstruction? | 25,1 | LLR and RLR have been reported in the setting of liver resection requiring biliary reconstruction. The technical difficulty and conversion rate should be acknowledged. | 1B |
|  |  | 25,2 | RLR facilitates fine suturing for bilioenteric anastomosis. | 1B |
| 26 | What is the role of LLR and RLR in the setting of liver resection requiring combined vascular (venous/arterial) reconstruction? | 26,1 | LLR and RLR have been reported in the setting of liver resection requiring combined vascular reconstruction. The technical difficulty should be acknowledged. | 2C |
|  |  | 26,2 | The robotic approach facilitates intracorporeal suturing. | 1C |

Supplementary Table 3. Domain 3 Clinical Questions and Recommendations

| **Q** | **Clinical Question** | **R** | **Recommendation** | **Code** |
| --- | --- | --- | --- | --- |
| 27 | Are there contraindications for laparoscopic liver resection (LLR), related to patient age? | 27 | Patient age should not be considered a contraindication for LLR. | 1B |
| 28 | Are there contraindications for robotic liver resection (RLR), related to patient age? | 28 | Patient age should not be considered a contraindication for RLR. | 1C |
| 29 | Are there contraindications for laparoscopic liver resection (LLR), related to obesity? | 29 | Obesity should not be considered a contraindication for LLR. | 1B |
| 30 | Are there contraindications for robotic liver resection (RLR), related to obesity? | 30 | There is limited evidence specifically addressing the impact of obesity on outcomes following RLR for different indications. Further studies are encouraged. | 1C |
| 31 | Are there contraindications for laparoscopic liver resection (LLR), related to complex previous abdominal operations? | 31 | Previous abdominal surgery should not be considered a contraindication for LLR. | 1B |
| 32 | Are there contraindications for robotic liver resection (RLR), related to complex previous abdominal operations? | 32 | Previous abdominal surgery should not be considered a contraindication for RLR. | 1C |
| 33 | Is LLR feasible in patients with large lesions? | 33 | LLR is feasible in patients with large lesions, when performed in centres with adequate experience and expertise. However, its technical complexity should be acknowledged. | 2B |
| 34 | Is RLR feasible in patients with large lesions? | 34 | RLR is feasible in patients with large lesions, when performed in centres with adequate experience and expertise. However, its technical complexity should be acknowledged. | 2B |
| 35 | Are LLR and RLR indicated for the treatment of (non-)colorectal liver metastases (CRLM/NCRLM) after neoadjuvant therapy? | 35 | Neoadjuvant therapy is not a contraindication for LLR or RLR in patients with CRLM/NCRLM. | 1C |
| 36 | Is LLR indicated for the treatment of patients with cirrhosis? | 36 | LLR is indicated in patients with Child-Pugh A and is associated with some advantages compared to open resection, including less liver-specific complications. Pushing boundaries beyond Child-Pugh A still needs further evidence. | 1A |
| 37 | Is RLR indicated for the treatment of patients with cirrhosis | 37 | RLR is indicated in patients with Child-Pugh A and is associated with some advantages compared to open resection, including less liver-specific complications. Pushing boundaries beyond Child-Pugh A still needs further evidence. | 1C |
| 38 | Is there a role for LLR in patients requiring 2-stage hepatectomy? | 38.1 | LLR could be considered in patients requiring a 2-stage hepatectomy, especially during the first stage. | 1C |
|  |  | 38.2 | LLR in second stage hepatectomy could be considered when performed in centres with adequate experience and expertise. | 2C |
| 39 | Is there a role for RLR in patients requiring 2-stage hepatectomy? | 39 | Although high-quality evidence is still lacking, RLR in 2-stage hepatectomy appears to be feasible in highly selected patients, when performed in centres with adequate experience and expertise. | 2C |
| 40 | Is there a role for LLR in patients requiring Portal Vein Embolization/Occlusion/Ligation (PVE/PVO/PVL)? | 40 | In selected patients, laparoscopic hepatectomy after portal vein embolization/occlusion/ligation is feasible and may be a valid alternative to the traditional open approach when performed in centres with adequate experience and expertise. | 2C |
| 41 | Is there a role for RLR in patients requiring Portal Vein Embolization/Occlusion/Ligation (PVE/PVO/PVL)? | 41 | Although no published evidence is currently available supporting the role of RLR after PVE/PVO/PVL, the robotic approach may achieve comparable outcomes to LLR. | 2C |
| 42 | Is there a role for LLR and RLR in patients requiring Liver Venous Deprivation (LVD)? | 42 | There is insufficient evidence to provide clear recommendations on the role of LLR and RLR in patients requiring LVD. Further research in this field should be promoted. | 1C |
| 43 | Is there a role for LLR in patients requiring ALPPS? | 43.1 | Laparoscopic ALPPS appears to be a feasible and safe procedure in highly selected cases and in experienced centres, even if its real advantages still need to be determined. | 2C |
|  |  | 43.2 | In the first stage of ALPPS the well-known benefits of laparoscopy could play an important role in improving patient outcomes. | 1C |
|  |  | 43.3 | Few data are available regarding the second stage of laparoscopic ALPPS, which appears feasible and safe in experienced hands, but should be limited to selected cases. | 2C |
| 44 | Is there a role for RLR in patients requiring ALPPS? | 44 | Robotic ALPPS represents a safe and feasible procedure, preserving the same advantages of the laparoscopic approach, when performed in centres with adequate experience and expertise. | 2C |
| 45 | Is LLR Feasible in patients with lesions in close proximity to major vessels? | 45 | LLR for lesions in proximity to major vessels is feasible, however its safety is dependent on the experience of the surgeon and the centre. | 2C |
| 46 | Is RLR Feasible in patients with lesions in close proximity to major vessels? | 46 | RLR for lesions in proximity to major vessels is feasible, however its safety is dependent on the experience of the surgeon and the centre. | 2C |
| 47 | Is LLR feasible in patients who underwent previous liver resection? | 47 | Laparoscopic repeated hepatectomy is feasible and safe with some short-term advantages in comparison to the traditional open approach. | 1B |
| 48 | Is RLR feasible in patients who underwent previous liver resection? | 48 | Robotic liver surgery appears feasible and safe in patients who have undergone previous hepatectomy. | 1C |

Supplementary Table 4. Domain 4 Clinical Questions and Recommendations

| **Q** | **Clinical Question** | **R** | **Recommendation** | **Code** |
| --- | --- | --- | --- | --- |
| 63 | Is laparoscopic liver resection (LLR) applicable for right and left hemihepatectomy? | 49.1 | LLR for RIGHT or LEFT hemihepatectomy is technically feasible and safe in terms of morbidity and oncological outcomes, with a shorter hospital stay and less blood loss but longer operative times compared with open surgery. | 1B |
|  |  | 49.2 | Laparoscopic surgery for right and extended right hemi hepatectomy remains a safe technique after modulation of the future liver remnant, although it is more complex and takes longer to perform than open surgery. | 2C |
| 64 | Is RLR applicable for RIGHT or LEFT hemihepatectomy? | 50 | Based on limited evidence, in centres with appropriate expertise, RLR for RIGHT or LEFT hemihepatectomy is technically feasible and safe in terms of morbidity and oncological outcomes, with a shorter hospital stay and less blood loss, but longer operative times compared with open surgery. Further studies are needed to confirm these findings. | 1C |
| 67 | Is LLR applicable for minor liver resections? | 51 | LLR is safe for minor liver resections and a minimally invasive approach should be the preferred approach in the anterolateral segments. | 1B |
| 68 | Is RLR applicable for minor liver resections? | 52 | Based on limited evidence, RLR appears feasible, safe and may be associated with lower conversion rates than LLR when adopted for minor liver resections in the anterolateral segments. | 2C |
| 69 | Is laparoscopic surgery applicable for Liver Resections in the 'Difficult Segments (1, 4a, 7, and 8)'? | 53 | LLR is technically feasible and safe for resections in the difficult segments and a minimally invasive approach is a valid alternative approach in centres with adequate experience and expertise. | 1B |
| 70 | Is RLR applicable for Liver Resections in the 'Difficult Segments (1, 4a, 7, and 8)'? | 54 | There is limited data on the feasibility and safety of RLR for liver resections in the difficult segments. Robotic articulation may facilitate surgery in this field. Future studies should focus on developing evidence in this specific field. | 2C |
| 71 | Is LLR applicable for parenchyma-sparing procedures? | 55.1 | When indicated, laparoscopic surgery for parenchyma-sparing procedures is technically feasible and safe in terms of short-term post-operative results and long-term oncological outcomes. This should be performed in centres with adequate experience and expertise. | 1B |
|  |  | 55.2 | There is limited evidence on the feasibility and oncological safety of laparoscopic surgery for multiple concomitant parenchyma-sparing resections, laparoscopic surgery should thus cautiously be considered in this setting. | 1B |
| 72 | Is RLR applicable for parenchyma-sparing procedures? | 56 | RLR for parenchyma-sparing procedures seems to be comparable to LLR with potential intraoperative benefits, but further data is needed. | 2C |
| 73 | Is LLR applicable for anatomic segmentectomies? | 57.1 | Laparoscopic surgery for anatomical monosegmentectomy appears to be safe and feasible. | 2B |
|  |  | 57.2 | Laparoscopic surgery for left lateral sectionectomy should be the preferred approach as it offers several perioperative benefits over open surgery, while perioperative costs and oncological outcomes are comparable. | 1B |
|  |  | 57.3 | Laparoscopic surgery for right posterior sectionectomy remains technically challenging but can offer perioperative benefits over open surgery in centres with adequate experience and expertise. | 2B |
|  |  | 57.4 | Evidence on the safety and feasibility of laparoscopic surgery for right anterior sectionectomy, central hepatectomy and segment 4b/5 bisegmentectomy is scarce, but favourable outcomes compared to open surgery have been reported. | 2B |
|  |  | 57.5 | Laparoscopic anatomical minor liver resection for hepatocellular carcinoma in patients with compensated cirrhosis livers is associated with lower postoperative morbidity rates than open liver resection. | 2B |
| 74 | Is RLR applicable for anatomic segmentectomies? | 58.1 | To date, there is no specific evidence on robot-assisted surgery for anatomical monosegmentectomy. Future studies should focus on developing evidence in this specific field. | 1C |
|  |  | 58.2 | Evidence for robotic right anterior sectionectomy or 4b/5 bisegmentectomy is limited and further studies are required. | 1C |

Supplementary Table 5. Domain 5 Clinical Questions and Recommendations

| **Q** | **Clinical Question** | **R** | **Recommendation** | **Code** |
| --- | --- | --- | --- | --- |
| 59 | What type of energy devices and instruments should be used during the parenchymal dissection phase in Laparoscopic Liver Resection (LLR)? | 59 | There are various instruments used for laparoscopic parenchymal dissection. There is not one instrument or device that appears to outperform others. | 1C |
| 60 | What type of energy devices and instruments should be used during the parenchymal dissection phase in Robotic Liver Resection (RLR)? | 60 | No agreement exists regarding the most valuable instrument to use for parenchymal transection during RLR. The development of such instruments is highly recommended. | 1C |
| 61 | What are the available techniques for parenchymal transection in LLR and RLR? | 61.1 | There are various techniques used for laparoscopic parenchymal dissection. There is not one technique that appears superior. Further research, ideally involving prospective or randomized studies, is needed to provide stronger recommendations. | 1C |
|  |  | 61.2 | There are various techniques used for robotic parenchymal dissection. There is not one technique that appears superior. Further research, ideally involving prospective or randomized studies, is needed to provide stronger recommendations. | 1C |
| 62 | Is there still a role for the hand-assisted technique and hybrid procedures for LLR? | 62 | Hand-assisted LLR or combined approaches are preferred to open surgeries with large incisions and can reduce conversion rates to open surgery in difficult resections, especially during the learning curve. | 1C |
| 63 | What is the role of intraoperative ultrasound in LLR? | 63.1 | Laparoscopic ultrasound should be available and considered in every case of LLR. Surgeons should be experienced in performing, interpreting and determining when intra-operative ultrasound is needed. | 1A |
|  |  | 63.2 | Laparoscopic ultrasound has been documented as a valuable tool for (1) confirming the diagnosis and staging of malignant lesions (2) assessing liver anatomy and planning resection plane, and (3) guidance during parenchymal transection. | 1B |
| 64 | What is the role of intraoperative ultrasound in RLR? | 64.1 | Intra-operative ultrasound should be available and considered in every case of RLR. Surgeons should be experienced in knowing the indications, performing and interpreting intra-operative ultrasound. | 1C |
|  |  | 64.2 | Both integrated robotic ultrasound and laparoscopic ultrasonography in RLR can be used. | 1C |
| 65 | What are the available safe techniques for inflow control during major anatomical resections in LLR? | 65 | Either the extrahepatic (hilar) or the Glissonean approaches are feasible and efficient laparoscopically; the choice between these two techniques is a matter of the surgeon’s preference. | 1B |
| 66 | What are the available safe techniques for inflow control during major anatomical resections in RLR? | 66 | Robotic extrahepatic (hilar) or Glissonean approaches are feasible and efficient; the decision of which technique is used for robotic right hemihepatectomy should be made based on the personal preferences and experience of the surgeon. | 1B |
| 67 | What are the available safe techniques during right hemihepatectomy in LLR? | 67.1 | Several surgical techniques are available in laparoscopic right hemihepatectomy, including different approaches (cranio-caudal/ventral-dorsal)and the additional use of the hanging manoeuvre. | 1B |
|  |  | 67.2 | The decision of which technique to use for laparoscopic right hemihepatectomy should be made based on the surgeon’s preferences and experience since none of these techniques have shown better outcomes. | 1B |
| 68 | What are the available safe techniques during right hemihepatectomy in RLR? | 68.1 | Several surgical techniques are available in robotic right hemihepatectomy, including different approaches (cranio-caudal/ventral-dorsal). | 1C |
|  |  | 68.2 | The decision of which technique is used for robotic right hemihepatectomy should be made based on the personal preferences and experience of the surgeon. | 1C |
| 69 | What are the available safe techniques during resection of the posterosuperior segments (segment 1, 4a, 7, 8) in LLR? | 69.1 | There are several techniques available during the resection of the posterosuperior segments in LLR, but the choice of any surgical technique should be made based on patient and tumour characteristics, as well as the surgeon’s preference and experience. | 1B |
|  |  | 69.2 | Patient positioning, right liver complete mobilization, and port placement play a key role during resection of the posterosuperior segments in LLR. | 1B |
| 70 | What are the available safe techniques during resection of the posterosuperior segments (segment 1, 4a, 7, 8) in RLR? | 70 | The choice of surgical technique should be made based on patient and tumour characteristics, as well as based on the preference and experience of the surgeon. | 1C |
| 71 | What are special techniques, tips and tricks to be adopted when performing a liver resection after future liver remnant modulation in LLR and RLR? | 71 | There are no standardized techniques during LLR and RLR after FLR modulation. Injection of ICG for the identification of the proper transection plane could be used. | 2C |
| 72 | What are special techniques, tips and tricks to be adopted when resecting lesions adherent to major vascular structures at critical distance (<1cm) from the hepatic hilum or IVC in LLR? | 72.1 | The use of energy device in proximity to major vessels, in particular close to the Glissonean pedicles should be used cautiously. Ultrasonic Surgical Aspiration System, crush-clamping technique and bipolar techniques are considered to be associated with lower risks. | 1C |
|  |  | 72.2 | The use of Pringle manoeuvre as well as the identification and isolation of Glissonean pedicles can be advantageous to minimize bleeding when resecting lesions close to the hepatic hilum. | 1C |
| 73 | When and How Should Conversions to Open Surgery Be Considered in LLR and RLR? | 73.1 | Consideration for early pre-emptive conversion to open surgery should be planned when intraoperative difficulties are expected. | 1C |
|  |  | 73.2 | In scenarios of uncontrolled bleeding, conversion is mandated. Specific institutional protocols for conversion should be developed when performing LLR/RLR. | 1C |
|  |  | 73.3 | Prompt decision-making for conversion, especially in situations of significant vascular injury, is crucial to reduce complications and enhance patient outcomes. | 2C |
|  |  | 73.4 | Before conversion it is important to try to gain temporary control of any active bleeding to avoid uncontrolled massive bleeding and gas embolism. | 1C |
|  |  | 73.5 | The decision-making process should be guided by individual patient factors, the extent of hepatic disease, tumour characteristics (in cancer surgery), and the surgeon’s experience, ensuring a tailored approach to each case. | 1C |
| 74 | What is the role of haemostatic products in minimally invasive liver surgery (MILS)? | 74 | In MILS the application of haemostatic products to the liver resection surface can be considered. | 1C |
| 75 | What are the optimal techniques for control of haemorrhage during LLR? | 75.1 | The use of Total or selective Hepatic Inflow Occlusion (TIO), Controlled Low Central Venous Pressure (CVP), stroke volume variation (SVV), and Mechanical Ventilation with Low Tidal Volume, are strongly recommended. | 1B |
|  |  | 75.2 | Intermittent Pringle manoeuvre is widely used, and it should be considered in case of inflow-related or transection-surface haemorrhage during LLR. | 1B |
| 76 | What are the optimal techniques for control of haemorrhage during RLR? | 76 | No strong recommendation are available due to the limited and low quality of available data. | 2C |
| 77 | Are there any specific recommendations on the use and positioning of drains in LLR and RLR, other than those known in the traditional open approach? | 77 | Routine prophylactic intra-abdominal drainage after LLR and RLR may not be necessary and may not affect postoperative outcomes. A case-by-case policy based on patient, procedure and specific factors may be adopted. | 1B |
| 78 | What are the recommended surgical techniques in LLR and RLR when performing a simultaneous colon and liver resection? | 78 | There are no specific recommendations regarding surgical techniques in LLR or RLR when performing a simultaneous colon and liver resection, hence the choice of surgical technique should be based on patient and tumour characteristics, as well as the surgeon’s preference and experience. | 1C |
| 79 | What are the recommended surgical techniques in LLR and RLR when performing a simultaneous rectal and liver resection? | 79 | There are no specific recommendations regarding surgical techniques in LLR or RLR when performing a simultaneous rectal and liver resection, hence the choice of surgical technique should be based on patient and tumour characteristics, as well as the surgeon’s preference and experience. | 1C |

Supplementary Table 6. Domain 6 Clinical Questions and Recommendations

| **Q** | **Clinical Question** | **R** | **Recommendation** | **Code** | **Comment** |
| --- | --- | --- | --- | --- | --- |
| 80 | Which parameters should be defined as core in the assessment of laparoscopic liver resection (LLR)? | 80 | intraoperative blood loss, operative time and hospital stay (days), conversion rate, R0/R1 resection rates and short-term clinical outcomes (mortality, overall complication rate, major complication rate (>grade 2 according to the Clavien-Dindo Classification), the Comprehensive Complication Index (CCI), failure to rescue rate each for 30 and 90 days after surgery) should be the core parameters in the assessment of LLR. | 1B |  |
| 81 | Which parameters should be defined as core in the assessment of robotic liver resection (RLR)? | 81.1 | Intraoperative blood loss, operative time and hospital stay (days), conversion rate, R0/R1 resection rates and short-term clinical outcomes (mortality, overall complication rate, major complication rate (>grade 2 according to the Clavien-Dindo Classification), the Comprehensive Complication Index (CCI), failure to rescue rate each for 30 and 90 days after surgery) should be the core parameters in the assessment of RLR. | 1B |  |
|  |  | 81.2 | Total costs should be defined as core in the assessment of RLR. | 1B | the validation committee strongly feels that total cost should be included, recognizing there are both direct and indirect costs involved with RLR |
| 82 | Which are the most suitable outcome measurements that can be used to assess the validity of LLR? | 82.1 | Composite outcome measures and benchmark values should be used to allow adequate comparison of the results. | 1B |  |
|  |  | 82.2 | A standardization of definitions for composite outcome measures specific to LLR is needed. | 1B |  |
| 83 | Which are the most suitable outcome measurements that can be used to assess the validity of RLR? | 83 | The parameters to assess validity of RLR should be the same as for LLR and open surgery. | 1C |  |
| 84 | What is the optimal outcome measurement we need to develop for future assessments in LLR and RLR? | 84.1 | Future assessments of LLR and RLR should integrate Patient-Reported Outcomes (PROs) using a minimal core set of Patient-Reported Outcome Measurements (PROMs). | 1C |  |
|  |  | 84.2 | This core set of PROMs needs to be defined in future research. | 1C |  |
|  |  | 84.3 | In patients who undergo surgery for cancer (CRLM or HCC), returning to oncologic therapy (measured by the delay between surgery and postoperative chemotherapy initiation, etc.) emerges as a relevant new measurement of postoperative outcome. | 2C |  |

Supplementary Table 7. Domain 7 Clinical Questions and Recommendations

| **Q** | **Clinical Question** | **R** | **Recommendation** | **Code** | **Comment** |
| --- | --- | --- | --- | --- | --- |
| 85 | What training and preparation should surgeons pursue before performing minor, major, and complex liver resections in LLR and RLR? | 85.1 | Implementing structured training programs with a step-up mastery progression is crucial, where advancement to the next phase is contingent upon. | 1C |  |
|  |  | 85.2 | Surgeons intending to start an LLR or RLR practice should first pursue training through fellowships, courses, and a proctoring program. | 1C |  |
|  |  | 85.3 | Undertaking major robotic hepatectomy without sufficient RLR experience is strongly discouraged. The learning curve typically involves a minimum of 25 robotic minor hepatectomies. | 1C |  |
|  |  | 85.4 | LLR experience is not essential prior to starting RLR; however, it may reduce the learning curve. | 1C |  |
| 86 | Who should be undertaking training and mentoring roles in LLR and RLR? | 86.1 | To ensure safe adoption of the technique, the roles of mentor and trainer should be undertaken by an expert liver surgeon who has completed their learning curve for complex LLR and/or RLR and has a track record in training. | 1C |  |
|  |  | 86.2 | Mentorship and/or proctorship in RLR by non-hepatobiliary robotic surgeons is discouraged and not appropriate in the advanced phase. | 1C |  |
| 87 | What is the impact of European and national training programs on clinical outcomes after LLR and RLR? | 87 | Structured training programs may help surgeons to safely start with LLR and RLR, acquire competency in a stepwise process, potentially lower learning curves and improve outcomes. | 1B |  |
| 88 | What is the role of proctorship in LLR and RLR training? | 88 | All surgeons without formal LLR and RLR training who intend to adopt LLR and RLR should receive proctoring in the initiation and expansion phases of their program. Surgeons are encouraged to have an in-person proctor for the initial learning curve of major/complex LLR and RLR. | 1C |  |
| 89 | What is needed to increase access to LLR and RLR training for practicing surgeons? | 89.1 | Implementation and increased availability of national and international structured training programs are recommended. | 1C |  |
|  |  | 89.2 | Incorporating LLR and RLR in HPB fellowships may increase the availability of LLR and RLR training for surgeons. | 1C |  |
|  |  | 89.3 | Increasing the availability of high-quality training materials such as surgical videos and technique descriptions is recommended. | 1C |  |
| 90 | Which centres should be performing LLR? | 90.1 | LLR programs should be established in centres with expertise in open liver surgery and considerations should include both volume-related and multidisciplinary structural elements. | 1B |  |
|  |  | 90.2 | While minor LLR procedures may yield comparable outcomes in both high and low-volume centres, complex and major procedures are advised to be managed in centres with adequate experience and expertise. | 1B |  |
|  |  | 90.3 | There is a significant volume-outcome relationship associated with better postoperative outcomes, lower mortality rates, improved failure-to-rescue rates, and improved oncological outcomes. | 1B |  |
| 91 | Which centres should be performing RLR? | 91.1 | RLR programs should be established in centres with expertise in open liver surgery and considerations should include both volume-related and multidisciplinary structural elements. | 2C |  |
|  |  | 91.2 | RLR should be performed in expert liver centres. LLR experience is not essential prior to starting RLR. However, experience with laparoscopic surgical procedures is recommended and may reduce the learning curve. | 1C |  |
| 92 | Should LLR be adopted in all liver surgical centres? | 92 | LLR is associated with improved short-term postoperative outcomes in selected patients. Therefore, it should be adopted in all liver surgery centres and offered to patients with the appropriate indications according to the local level of proficiency | 1A |  |
| 93 | Should RLR be adopted in all liver surgical centres? | 93 | At this moment, there is no evidence to support the adoption of RLR in all liver surgical centres. | 2C |  |
| 94 | What is the path for medium-volume centres to be able to offer LLR and RLR major hepatectomy safely? | 94 | Recommendations given previously regarding training and preparation for LLR and RLR should be regarded (topic 24). Additionally, collaboration with LLR and RLR referral centres, case observation, and proctorship could facilitate the safe adoption of major LLR and RLR in medium-volume centres.” | 1C |  |
| 95 | What should be the role of national and international registries in the wider implementation of minimally invasive liver surgery (MILS)? | 95.1 | The broader adoption of MILS should be advocated by national and international HPB associations. | 1B |  |
|  |  | 95.2 | HPB associations should actively endorse the establishment, implementation, and coordination of national registries, along with active participation in international registries. | 1B |  |
| 96 | Should centres be asked to include minimally invasive patients in registries for quality control and who should implement such guidelines? | 96.1 | Centres should be strongly encouraged to include MILS patients in registries for quality control. | 1B |  |
|  |  | 96.2 | Collaboration among medical organizations, professional societies, and healthcare authorities is crucial for implementing guidelines that standardize data collection and improve quality control in MILS. | 1B |  |
| 97 | Is LLR associated with higher costs than the traditional open approach? | 97 | The higher operative costs in LLR are offset by lower hospitalization costs (due to shorter hospital stay and less overall complications) compared to OLR, leading to no statistically significant difference in total costs in a RCT and a systematic review with economic analysis. | 1A |  |
| 98 | Is RLR associated with higher costs than the traditional open approach? | 98 | The available data is inconclusive about the true overall costs of RLR compared to the open approach with some studies showing cost advantages and others showing cost disadvantages.” High-level evidence is needed to estimate the true cost-effectiveness of one approach over the other.” | 1B |  |
| 99 | Are the additional costs of LLR justified? | 99 | In view of reduced length of stay, time to recovery, time to adjuvant therapy, time to return to work, and potentially reduced overall complications, the higher operative costs are justified for LLR. | 1A |  |
| 100 | Are the additional costs of RLR justified? | 100 | Based on current data, there is little evidence to justify the additional costs associated with RLR compared to LLR. Further evidence is recommended. | 1C |  |
| 101 | How should additional costs of LLR be mitigated? | 101 | Sufficient centre volume and rigorous utilization of enhanced recovery programs with early discharge can potentially mitigate additional procedural costs of LLR. | 1B |  |
| 102 | How should additional costs of RLR be mitigated? | 102 | Extended life programs for robotic instruments and increased competition in the market should be encouraged to decrease costs for RLR. | 2C | the validation committee strongly recommends adding this recommendation to the guidelines. After a revote, it passed with 97% and was added. |
| 103 | Is LLR more sustainable (lower carbon footprint) than RLR? | 103 | Currently, there is not enough evidence to favour one technique over the other in terms of sustainability in liver surgery. In cases where there is no clinical benefit for one approach over the other, surgeons are encouraged to opt for the approach with the least waste and lower carbon footprint. | 1C |  |
| 104 | What is needed to increase access to RLR? | 104 | Increased access to cost-efficient robotic systems along with systematic training programs for RLR (see recommendations on implementation and training) are essential to improve access. | 2C |  |

Supplementary Table 8. Domain 8 Clinical Questions and Recommendations

| **Q** | **Clinical Question** | **R** | **Recommendation** | **Code** |
| --- | --- | --- | --- | --- |
| 105 | What is the role of radiomics in minimally invasive liver surgery (MILS)? | 105.1 | Radiomic features extracted from preoperative imaging modalities such as MRI and CT should be used because of its ability to provide valuable information about prognosis, diagnosis and treatment of liver malignancy. | 2C |
|  |  | 105.2 | Further validation studies are still needed to confirm the utility of radiomics analysis in clinical practice. | 1C |
| 106 | Is 3D liver reconstruction essential and in which cases should it be used? | 106.1 | 3D liver reconstruction should play a crucial role in treatment planning and intraoperative decision-making because it has the potential to improve clinical decision-making and reduce blood loss and operative time. | 1B |
|  |  | 106.2 | 3D liver reconstruction should be particularly encouraged in patients with liver mass adjacent to the major vascular structures of the liver and/or in patients planned to undergo major hepatectomy, and in patients planned to undergo anatomical segmentectomy. | 1B |
|  |  | 106.3 | If available, the integration of 3D liver reconstruction technology should be prioritized into the education and training of junior surgeons, fellows, residents, and students due to its potential to significantly enhance the understanding of liver anatomy, tumour location, and the planning of surgical interventions. | 1C |
|  |  | 106.4 | If available, the use of CT/MRI-based 3D liver reconstruction technology should be encouraged because it may provide a superior understanding of liver anatomy, and augment the visual information for the surgeon, and ensure that everyone involved in the operation is on the same page regarding the resection that is planned. | 1C |
| 107 | Who should do 3D liver reconstruction? | 107.1 | The hepatobiliary surgeons should be a key component in 3D liver reconstruction process because they decide the ultimate operative plan. Radiologists should still be primarily responsible for identifying liver lesions, tumours and vascular structures. | 1C |
|  |  | 107.2 | Medical imaging specialists with expertise in advanced visualization and 3D reconstruction techniques should be increasingly encouraged for the technical aspects of generating high-quality 3D liver reconstructions. | 1C |
|  |  | 107.3 | The role of radiology and medical imaging specialists should be expected to diminish as programs that can generate 3D images from cross-sectional images continue to develop autonomous functionality, potentially eliminating the necessity for direct involvement of a radiologist. | 2C |
| 108 | What are the purposes for 3D liver reconstruction use pre- and intraoperatively? | 108.1 | In the future optimal surgical planning for liver tumours should include 3D liver reconstruction preoperatively. | 1B |
|  |  | 108.2 | liver reconstruction may facilitate pre-surgical planning by better showing the relation of the tumour mass with adjacent anatomical structures. | 1C |
|  |  | 108.3 | 3D reconstructed models should be utilized intraoperatively, if available, because they have been shown to provide better navigation in difficult areas by allowing surgeons to better anticipate anatomical issues that may arise during surgery. | 1C |
| 109 | Is liver segmentation essential and in which cases should it be used? | 109 | Liver segmentation should be used, in particular, for parenchymal sparing liver resection and in patients with marginal liver function/condition (e.g. cirrhotic, CASH, NASH, steatotic liver). | 1C |
| 110 | Who should do liver segmentation? | 110 | The task of liver segmentation should primarily be undertaken by a multidisciplinary team consisting of hepatobiliary surgeons, radiologists, and medical imaging specialists. | 1C |
| 111 | What are the purposes for the use of liver segmentation pre- and intra-operatively? | 111.1 | Preoperative liver segmentation can serve a fundamental component of surgical planning by enabling accurate volumetry and visualization of liver segments. It can also play a vital role in defining vessel and tumour relationship, which can provide valuable information for clinical decision-making and treatment strategies. | 2C |
|  |  | 111.2 | Intraoperative liver segmentation should be prioritized during minimally invasive anatomical liver resection because accurate segmentation is necessary to accomplish precise pedicle control and parenchymal resection. | 1C |
|  |  | 111.3 | Liver segmentation should be promoted for optimal image-guided surgery by maximizing accurate and precise operations through a combination of preoperative planning and intraoperative image-guided liver surgery systems. | 2C |
| 112 | Is there a role in MILS for augmented reality in soft tissue structures like the liver, that are deformable and malleable? | 112.1 | Augmented reality should be promoted because of its potential to improve surgical outcomes in liver surgery during both minimally invasive and open liver procedures. However, further research should be promoted to evaluate the long-term impact of AR on clinical outcomes and to optimize the accuracy and reliability of AR systems. | 2C |
|  |  | 112.2 | needs ongoing and further development for deformable and malleable organs such as the liver to provide a dynamic reconstruction that can adjust for changes in tissue shape and orientations as the operation progresses. | 1C |
| 113 | Can artificial intelligence (AI) help in pre-operative risk assessment and in nutritional status assessment in patients undergoing MILS? | 113 | Although there is currently no evidence demonstrating AI's ability to determine nutritional status specifically for patients undergoing MILS, research in this area should be encouraged due to the potential benefits. | 1C |
| 114 | What role should MILS surgeons play in pushing forward the role of AI in their daily practice? | 114.1 | MILS surgeons should play a proactive role in embracing and leveraging advanced technologies, including robotic systems equipped with AI, to enhance surgical techniques and outcomes. | 1C |
|  |  | 114.2 | Surgeons should play a crucial role in driving forward the integration of AI in their daily practice by actively engaging in collaborative initiatives, embracing advanced technologies such as robotic surgery, and exploring the potential benefits of AI in surgery and patient care. | 1C |
|  |  | 114.3 | er surgeons have important roles in creating collaboration with industry to help develop AI technology (e.g. Liver surgeons can share CT scans of liver tumours with industry to use for development of AI machine learning, so the software can automatically recognize the organ/structures from scans). | 1C |
|  |  | 114.4 | Surgeons should also give constructive feedback of the accuracy of AI systems and how to increase their usefulness in clinical practice. | 1C |
| 115 | What is the role of AI in the future expansion of laparoscopic liver resection (LLR)? | 115 | AI should be encouraged because it has the potential to enhance minimally invasive hepatic surgery by improving precision, safety, and accessibility for patients worldwide. | 2C |
| 116 | What is the role of AI in the future expansion of robotic liver resection (RLR)? | 116 | Liver surgeons should begin preparing now for computer-assisted surgery to be widely equipped with AI in the future. | 1C |
| 117 | What is the role of autonomous actions in minimally invasive liver surgery? | 117 | Autonomous actions during minimal invasive liver surgery should be studied because of their potential to be associated with a reduction in intraoperative complications and overall improvement of postoperative outcomes by reducing stress and fatigue for the surgeon during the operation. | 2C |
| 118 | Is there a role for telemanipulation? | 118.1 | Telemanipulation should be incorporated into the armamentarium of liver surgeons because it has the potential to enhance manipulation and dexterity during surgery and the ability to suppress resting tremors. | 2C |
|  |  | 118.2 | Telemanipulation should be encouraged because it can permit telesurgery. Legal barriers to telesurgery should be addressed in a multidisciplinary fashion and prioritized because it has the potential to be extremely useful for real time intraoperative mentoring of remote surgeons. | 2C |
| 119 | Is there a role for handheld robotics? | 119.1 | Handheld robotics should be studied because it has the advantage of maintaining haptics. | 2C |
|  |  | 119.2 | Due to financial constraints, the further development of handheld robotics should be encouraged to ensure that the maximal number of patients can benefit from robotics worldwide. | 2C |
| 120 | Is there a role for non-console robotic systems? | 120 | Non-console robots may have potential safety benefits because the surgeon can stay at the bedside throughout the procedure. | 2C |
| 121 | Is indocyanine green (ICG) essential in LLR and in which cases should it be used? | 121 | The usage of indocyanine green (ICG) should be further studied because it exhibits variability with regard to indications and may help during identification of intersegmental planes. | 1C |
| 122 | Is ICG essential in RLR and in which cases should it be used? | 122 | ICG's existence in the current robotic surgery platform, its accessibility and ease of application, and the improved intraoperative and postoperative results it provides support the fact that it should be used routinely for robotic liver surgery. | 2C |
| 123 | How should ICG be used in LLR? | 123 | ICG should be administered peripherally or via direct injection into the portal vein, with dosages tailored to the desired indication. | 1C |
| 124 | How should ICG be used in RLR? | 124 | The administration of ICG in RLR should be done intravascularly, whether peripherally or via direct injection into the portal vein, with dosages tailored to the timing thereof. | 1C |
| 125 | Should negative or positive ICG staining be used in LLR? | 125.1 | Both negative and positive ICG staining can be employed. | 1C |
|  |  | 125.2 | Negative staining should be utilized for : 1. Patients scheduled to undergo anatomical liver resection via the Glissonean approach 2. When employing selective clamping of the pedicle, followed by intravenous injection of indocyanine green 3. Cases of portal vein embolization or tumour invasion that completely occludes the targeted portal vein. | 2C |
|  |  | 125.3 | The positive staining method should be favoured mostly in the context of anatomical resections in the posterosuperior segments and needs more surgical experience. More studies are needed to determine the superiority of each technique and optimal indications for their application. | 2C |
| 126 | Should negative or positive ICG be used in RLR? | 126.1 | Both negative and positive ICG staining can be employed. | 1C |
|  |  | 126.2 | Negative staining should be utilized for : 1. Patients scheduled to undergo anatomical liver resection via the Glissonean approach 2. When employing selective clamping of the pedicle, followed by intravenous injection of indocyanine green 3. Cases of portal vein embolization or tumour invasion that completely occludes the targeted portal vein. | 2C |
|  |  | 126.3 | The positive staining method should be favoured mostly in the context of anatomical resections in the posterosuperior segments and needs more surgical experience. More studies are needed to determine the superiority of each technique and optimal indications for their application. | 2C |
| 127 | What is the ideal timing of preoperative administration of ICG in LLR? | 127.1 | The widely acknowledged practice should be the administration of ICG between the 2nd and 14th days preceding the surgical procedure. | 1C |
|  |  | 127.2 | Optimal timing for application falls within the second period (7-14 days) in cases of cirrhosis and first period (2-7 days) in case of no liver parenchymal diseases. | 1C |
|  |  | 127.3 | If ICG is used to help visualize the bile duct intraoperatively, it is given 30-45 minutes earlier to allow enough time for ICG to be excreted and present in the biliary system. | 1C |
| 128 | What is the ideal timing of preoperative administration of ICG in RLR? | 128.1 | The widely acknowledged practice should be the administration of ICG between the 2nd and 14th days preceding the surgical procedure. | 1C |
|  |  | 128.2 | Optimal timing for application falls within the second period (7-14 days) in cases of cirrhosis and first period (2-7 days) in case of no liver parenchymal diseases. | 1C |
|  |  | 128.3 | If ICG is used to help visualize the bile duct intraoperatively, then it should be given 30-45 minutes earlier to allow enough time for ICG to be excreted and present in the biliary system. | 1C |
| 129 | Are there differences in ICG administration and interpretation based on liver parenchyma status in LLR? | 129 | Diffuse staining patterns and false positivity can occur in cases of cirrhosis, parenchymal liver illnesses, and disorders associated with bile excretion from the liver, as a result, the timing of preoperative administration should be extended in order to guarantee precise findings while used for tumour identification. | 1C |
| 130 | Are there differences in ICG administration and interpretation based on liver parenchyma status in RLR? | 130 | Diffuse staining patterns and false positivity can occur in cases of cirrhosis, parenchymal liver illnesses, and disorders associated with bile excretion from the liver; as a result, the timing of preoperative administration should be extended in order to guarantee precise findings while used for tumour identification. | 1C |
| 131 | Is liver navigation essential in LLR and in which cases should it be used? | 131.1 | The integration of liver navigation may offer real-time visualization, accurate tumour localization, precise imaging of liver segments, and improved spatial orientation. | 2C |
|  |  | 131.2 | The utilization of liver navigation could be particularly beneficial during major hepatectomy, technically complex resections, and anatomical liver resection. | 2C |
| 132 | Is liver navigation essential in RLR and how should it be used? | 132.1 | The integration of liver navigation may offer real-time visualization, accurate tumour localization, precise imaging of liver segments, and improved spatial orientation. | 2C |
|  |  | 132.2 | The utilization of liver navigation could be particularly beneficial during major hepatectomy, technically complex resections, and anatomical liver resection. | 2C |
| 133 | How should liver navigation be used in LLR? | 133 | Liver navigation should be considered as the utilization of intraoperative ultrasound guidance, fluorescence image guidance, virtual reality technology, and/or 3D reconstruction for real-time visualization to improve the accuracy and safety of surgical procedures. | 2C |
| 134 | How should liver navigation be used in RLR? | 134 | Liver navigation should be considered as utilization of intraoperative ultrasound guidance, fluorescence image guidance, virtual reality technology and/or 3D reconstruction for real time visualization to improve accuracy and safety of surgical procedure. | 2C |
| 135 | Is haptic feedback currently available in RLR? | 135 | The lack of haptic feedback is a limitation of currently available robotic console systems. | 2C |
| 136 | Is haptic feedback essential in RLR? | 136 | Although haptic feedback plays a crucial role in open and laparoscopic surgical precision, tissue manipulation, and assessment of tissue properties and detection of intrahepatic liver lesions, this does not seem to be the case for current RLR. | 2C |
| 137 | What is needed for the development of haptic feedback in RLR? | 137 | The potential benefits of haptic feedback should be further developed. | 2C |

Supplementary Table 9. Agreement Rates and Topic Mean Performance Scores

|  | **Topic 1** | **Topic 2** | **Topic 3** | **Topic 4** | **Topic 5** | **Topic 6** | **Topic 7** | **Topic 8** | **Topic 9** | **Topic 10** | **Topic 11** | **Topic 12** | **Topic 13** | **Topic 14** |
| --- | --- | --- | --- | --- | --- | --- | --- | --- | --- | --- | --- | --- | --- | --- |
| **Item 1** | 72.2% | 70.0% | 73.9% | 80.0% | 72.2% | 79.4% | 71.1% | 73.3% | 64.4% | 62.8% | 62.2% | 56.7% | 54.4% | 57.8% |
| **Item 2** | 73.3% | 70.0% | 74.4% | 80.6% | 72.2% | 79.4% | 68.9% | 72.8% | 62.2% | 61.1% | 57.8% | 56.7% | 55.0% | 58.3% |
| **Item 3** | 71.7% | 68.9% | 71.7% | 78.9% | 70.6% | 78.3% | 70.0% | 72.8% | 61.1% | 61.7% | 58.9% | 54.4% | 51.1% | 58.3% |
| **Item 4** | 73.3% | 71.1% | 73.9% | 78.3% | 69.4% | 78.9% | 68.9% | 74.4% | 60.6% | 58.9% | 56.7% | 55.6% | 53.3% | 58.9% |
| **Item 5** | 72.8% | 69.4% | 73.9% | 79.4% | 71.1% | 79.4% | 70.6% | 74.4% | 61.7% | 60.0% | 57.2% | 57.2% | 55.0% | 58.9% |
| **Item 6** | 73.3% | 72.8% | 75.6% | 80.0% | 75.0% | 81.1% | 70.0% | 77.2% | 56.7% | 52.2% | 56.1% | 56.7% | 54.4% | 58.9% |
| **Item 7** | 73.3% | 72.8% | 75.0% | 79.4% | 73.3% | 78.3% | 70.0% | 77.8% | 57.2% | 52.2% | 55.6% | 56.7% | 55.0% | 58.9% |
| **Mean Performance score (per topic)** | **72.8%** | **70.7%** | **74.1%** | **79.5%** | **72.0%** | **79.3%** | **69.9%** | **74.7%** | **60.6%** | **58.4%** | **57.8%** | **56.3%** | **54.0%** | **58.6%** |

| **Topic 15** | **Topic 16** | **Topic 17** | **Topic 18** | **Topic 19** | **Topic 20** | **Topic 21** | **Topic 22** | **Topic 23** | **Topic 24** | **Topic 25** | **Topic 26** | **Topic 27** | **Mean Performance Score (per item)** |
| --- | --- | --- | --- | --- | --- | --- | --- | --- | --- | --- | --- | --- | --- |
| 57.2% | 47.2% | 48.9% | 50.0% | 50.0% | 56.1% | 50.6% | 48.3% | 47.8% | 41.1% | 40.6% | 41.7% | 37.2% | ***58.0%*** |
| 54.4% | 46.1% | 47.8% | 50.6% | 50.0% | 55.0% | 48.9% | 47.8% | 45.6% | 40.6% | 40.0% | 40.6% | 36.7% | ***57.3%*** |
| 57.8% | 46.1% | 48.9% | 51.1% | 49.4% | 56.1% | 48.9% | 47.8% | 47.2% | 41.1% | 40.6% | 41.7% | 36.7% | ***57.1%*** |
| 56.1% | 46.7% | 49.4% | 50.0% | 50.0% | 55.0% | 49.4% | 47.8% | 46.1% | 40.6% | 40.0% | 41.7% | 37.2% | ***57.1%*** |
| 56.1% | 47.8% | 49.4% | 52.2% | 51.1% | 55.6% | 50.0% | 47.8% | 47.2% | 40.6% | 41.1% | 42.2% | 37.2% | ***57.8%*** |
| 55.6% | 47.2% | 50.6% | 52.2% | 53.3% | 57.8% | 50.6% | 50.6% | 48.3% | 41.1% | 40.0% | 41.7% | 37.8% | ***58.0%*** |
| 56.1% | 47.8% | 50.6% | 52.8% | 53.9% | 58.3% | 50.6% | 50.6% | 48.3% | 42.2% | 39.4% | 42.2% | 37.2% | ***58.0%*** |
| **56.2%** | **47.0%** | **49.4%** | **51.3%** | **51.1%** | **56.3%** | **49.9%** | **48.7%** | **47.2%** | **41.0%** | **40.2%** | **41.7%** | **37.1%** | **57.6%** |
